# The proteomic landscape of soft tissue sarcomas

**DOI:** 10.1038/s41467-023-39486-2

**Published:** 2023-06-29

**Authors:** Jessica Burns, Christopher P. Wilding, Lukas Krasny, Xixuan Zhu, Madhumeeta Chadha, Yuen Bun Tam, Hari PS, Aswanth H. Mahalingam, Alexander T. J. Lee, Amani Arthur, Nafia Guljar, Emma Perkins, Valeriya Pankova, Andrew Jenks, Vanessa Djabatey, Cornelia Szecsei, Frank McCarthy, Chanthirika Ragulan, Martina Milighetti, Theodoros I. Roumeliotis, Stephen Crosier, Martina Finetti, Jyoti S. Choudhary, Ian Judson, Cyril Fisher, Eugene F. Schuster, Anguraj Sadanandam, Tom W. Chen, Daniel Williamson, Khin Thway, Robin L. Jones, Maggie C. U. Cheang, Paul H. Huang

**Affiliations:** 1 Division of Molecular Pathology, The Institute of Cancer Research, London, UK; 2 Division of Clinical Studies, The Institute of Cancer Research, London, UK; 3 The Royal Marsden NHS Foundation Trust, London, UK; 4 Division of Cancer Biology, The Institute of Cancer Research, London, UK; 5 Wolfson Childhood Cancer Research Centre, Translational and Clinical Research Institute, Newcastle University Centre for Cancer, Newcastle University, Newcastle upon Tyne, UK; 6 Leeds Institute of Medical Research at St James’s, St James’s University Hospital, Leeds, UK; 7 University Hospitals Birmingham NHS Foundation Trust, Birmingham, UK; 8 Ralph Lauren Centre for Breast Cancer Research, The Royal Marsden NHS Foundation Trust, London, UK; 9 Division of Breast Cancer Research, The Institute of Cancer Research, London, UK; 10 Department of Oncology, National Taiwan University Hospital, Taipei City, Taiwan; 11 Graduate Institute of Oncology, National Taiwan University College of Medicine Taipei, Taipei City, Taiwan

**Keywords:** Sarcoma, Proteomic analysis, Sarcoma, Cancer genomics, Tumour biomarkers

## Abstract

Soft tissue sarcomas (STS) are rare and diverse mesenchymal cancers with limited treatment options. Here we undertake comprehensive proteomic profiling of tumour specimens from 321 STS patients representing 11 histological subtypes. Within leiomyosarcomas, we identify three proteomic subtypes with distinct myogenesis and immune features, anatomical site distribution and survival outcomes. Characterisation of undifferentiated pleomorphic sarcomas and dedifferentiated liposarcomas with low infiltrating CD3 + T-lymphocyte levels nominates the complement cascade as a candidate immunotherapeutic target. Comparative analysis of proteomic and transcriptomic profiles highlights the proteomic-specific features for optimal risk stratification in angiosarcomas. Finally, we define functional signatures termed Sarcoma Proteomic Modules which transcend histological subtype classification and show that a vesicle transport protein signature is an independent prognostic factor for distant metastasis. Our study highlights the utility of proteomics for identifying molecular subgroups with implications for risk stratification and therapy selection and provides a rich resource for future sarcoma research.

## Abstract (web summary)

Characterising the molecular profile of soft tissue sarcomas (STS) remains critical. Here, the authors analyse samples from 321 STS patients across 11 histological subtypes using proteomics and identify prognostic signatures that can be applied to multiple subtypes.

## Introduction

Soft tissue sarcomas (STS) are a group of rare and diverse mesenchymal malignancies comprising more than 80 histological subtypes^1^. At the genomic level, these tumours fall into two main categories, those with complex karyotypes or those with specific genetic alterations such as translocations and point mutations^2^. However, the biological understanding of these disparate diseases remains incomplete due in part to the inherent molecular heterogeneity within and between histological subtypes. Clinical management of localised disease is dependent on anatomical site, tumour grade and histological subtype^3–6^ but despite multi-disciplinary management, cure rates in the localised setting remain unsatisfactory with up to 50% of patients developing tumour relapse after surgery^7–9^. Following recurrence, patients with locally advanced and metastatic STS have poor outcomes with limited systemic treatment options^10,11^. To improve patient outcomes, there is a need to move away from current “one size fits all” treatment approaches towards molecular strategies able to dissect the biological heterogeneity inherent in STS, and deliver better risk stratification tools and biomarker-matched therapies.

While several large-scale genomic and epigenomic pan-STS studies have been published^12–15^, these findings have yet to be translated into routine clinical management. Proteins are key mediators of cellular communication and serve as targets for multiple oncology drugs and ancillary diagnostic tests^16,17^. Proteomics is thus complementary to genomic studies and could bridge this translational gap. Underscoring the importance of protein-level analysis, recent large-scale proteomic profiling studies from The Clinical Proteomic Tumour Analysis Consortium (CPTAC) in multiple epithelial cancer types have led to improved molecular classification beyond what can be achieved by genomic or transcriptomic data alone, with the identification of cancer drivers and biomarkers with clinical utility^18–22^.

A pan-STS proteomic study was reported by The Cancer Genome Atlas (TCGA) consortium using the Reverse Phase Protein Array (RPPA) platform to profile 206 cases across 6 histological subtypes^12^. However, RPPA is a targeted platform that is limited to a few hundred pre-selected proteins and unlike mass spectrometry (MS)-based analysis does not provide a systems-level view of the proteome^23^. Furthermore, prior STS molecular profiling studies have relied on fresh frozen material which, in contrast to formalin-fixed paraffin-embedded (FFPE) specimens, is typically not archived in biobanks. Molecular profiling of adequate numbers of frozen specimens with sufficient follow-up for long-term survival analysis is often impractical for rare cancers and therefore large-scale proteomic studies require methods that are compatible with standard FFPE tissue.

Here we present the proteomic landscape of STS comprising a well-annotated cohort of FFPE specimens from 321 cases spanning 11 histological subtypes, including paediatric and adult patients, across multiple anatomical sites. Undertaking histological subtype-specific, immune-based and pan-sarcoma analyses, we show the utility of this MS-based proteomic resource in addressing the biological heterogeneity in STS by revealing defined molecular subgroups with implications for clinical risk stratification and selection of therapy.

## Results

### Samples and clinicopathological data

**Fig. 1:**
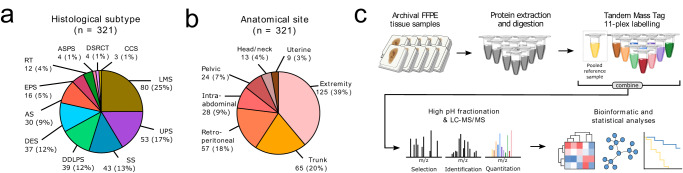
Schematic overview of the study. **a**, **b** Pie charts showing the count and percentage breakdown of histological subtypes (**a**) and anatomical sites (**b**) within the study cohort. **c** Overview of the proteomic analysis workflow. AS angiosarcoma, ASPS alveolar soft part sarcoma, CCS clear cell sarcoma, DDLPS dedifferentiated liposarcoma, DES desmoid tumour, DSRCT desmoplastic small round cell tumour, EPS epithelioid sarcoma, LMS leiomyosarcoma, RT rhabdoid tumour, SS synovial sarcoma, UPS undifferentiated pleomorphic sarcoma, FFPE formalin-fixed paraffin-embedded, LC liquid chromatography, MS mass spectrometry.

The cohort is comprised of a multi-institutional series of 321 primary cases (Fig. 1a) including the more common STS histological subtypes such as leiomyosarcoma (LMS), undifferentiated pleomorphic sarcoma (UPS), synovial sarcoma (SS), dedifferentiated liposarcoma (DDLPS), as well as rare and ultra-rare sarcoma subtypes of angiosarcoma (AS), epithelioid sarcoma (EPS), extracranial rhabdoid tumour (RT), alveolar soft part sarcoma (ASPS), desmoplastic small round cell tumour (DSRCT) and clear cell sarcoma (CCS). Among these subtypes, four are known to have complex karyotypes (LMS, UPS, DDLPS, AS) with the remainder harbouring simple genomes characterised either by translocations (SS, ASPS, DSRCT, CCS), or loss of SWI/SNF complex components (EPS, RT). Desmoid tumours (DES), a locally aggressive soft tissue neoplasm that lacks metastatic potential and harbours *CTNNB1* mutations have also been included in the cohort. The median age at diagnosis was 58.4 (range: 0.1–90) years and most cases were intermediate to high grade (79%) (full clinicopathological information provided in Supplementary Data 1). All specimens included in this study are comprised of primary tumours which were resected from a range of anatomical locations with extremity cases being the most common site (Fig. 1b). Of the cases included in this study, 40 patients (12%), the majority of which were SS (*n* = 25), had undergone pre-operative therapy (Supplementary Data 1).

### Pan-sarcoma proteomic landscape analysis

The workflow of the proteomic pipeline is outlined in Fig. 1c. FFPE tissue was reviewed for tumour cell content and subjected to protein extraction and digestion. A multiplexed isobaric labelling strategy (Tandem Mass Tag 11-plex) was utilised where 10 unique cases were analysed in every MS run with the 11th sample being a pooled reference from multiple cases representative of the diversity of STS subtypes within the cohort. This pooled reference enabled normalisation across the entire dataset of 321 cases for further downstream analysis.

**Fig. 2:**
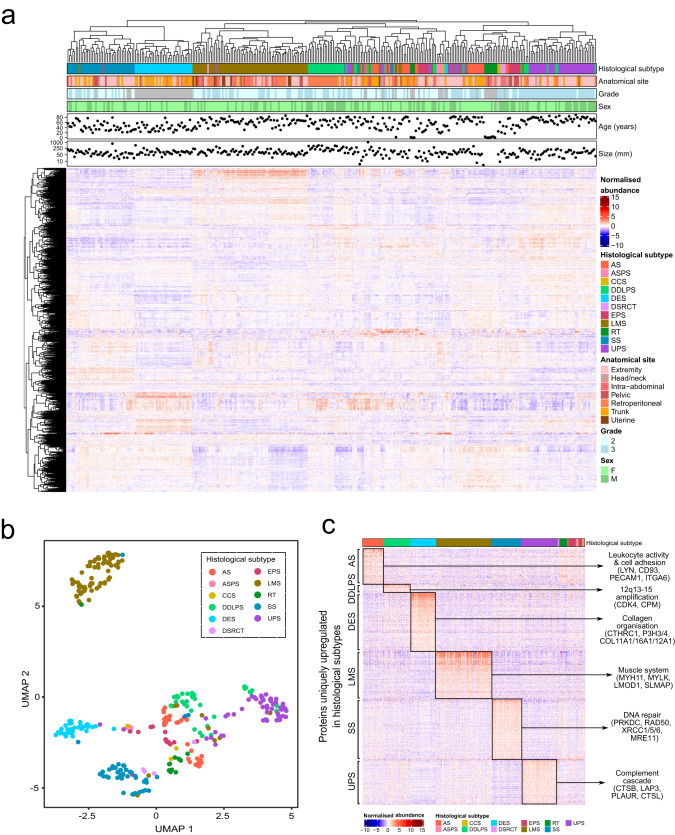
The proteomic landscape of soft tissue sarcoma. **a** Annotated heatmap showing the unsupervised clustering (Pearson’s distance) of 3290 proteins across the study cohort. From top to bottom, panels indicate histological subtype, anatomical site, tumour grade, patient sex, patient age, and tumour size. **b** Uniform manifold approximation and projection (UMAP) of the proteomic data with individual cases coloured by histological subtype. **c** Heatmap showing the proteins (*n* = 1362) uniquely upregulated in histological subtypes with greater than 20 cases in the cohort (FDR < 1%, fold change ≥1.5), sorted by histology. Annotations indicate key proteins (DDLPS & SS) identified by significant analysis of microarray (SAM) and gene sets (AS, DES, LMS, UPS) identified by overrepresentation analysis in each histological subtype (Supplementary Data 4). AS angiosarcoma, ASPS alveolar soft part sarcoma, CCS clear cell sarcoma, DDLPS dedifferentiated liposarcoma, DES desmoid tumour, DSRCT desmoplastic small round cell tumour, EPS epithelioid sarcoma, LMS leiomyosarcoma, RT rhabdoid tumour, SS synovial sarcoma, UPS undifferentiated pleomorphic sarcoma.

A total of 8148 proteins were identified with 3290 proteins quantified across all samples (Supplementary Data 2), with an average of 4313 proteins identified per MS experiment. A subset of cases where duplicate sample extractions from the same tumour block were analysed showed high reproducibility with a mean Pearson’s correlation coefficient of *r* = 0.81. Unsupervised clustering of the proteins quantified across all samples indicates that LMS, DES and SS each had a unique proteomic profile with cases clustering by histological subtype and not anatomical site (Fig. 2a). Uniform manifold approximation and projection for dimension reduction (UMAP) analysis highlight LMS as the most distinct sarcoma subtype in the cohort based on proteomic profiles (Fig. 2b). Overrepresentation analysis of proteins that are exclusively upregulated in each subtype (with at least 20 cases) highlighted key biological processes and proteins that operate in these subtypes (Fig. 2c, Supplementary Data 3 and 4).

Although no ontologies were enriched in the overrepresentation analysis of DDLPS, consistent with the amplification of *CDK4* in a large proportion of DDLPS^24^, our data shows that at the protein level, CDK4 is highly expressed in this subtype (Fig. S1A and Supplementary Data 3). Upregulated LMS proteins were predominantly composed of muscle system ontologies which are reflective of the smooth muscle lineage of this histological subtype^25^. Of the proteins that were identified as significantly upregulated in LMS, three proteins (MYH11, SRC and GAPDH) were also present in the RPPA dataset from the independent TCGA sarcoma cohort (LMS *n* = 80, SS *n* = 10, DDLPS *n* = 50, UPS *n* = 44)^12^. Evaluation of the expression levels of these proteins in the TCGA cohort finds that they are similarly upregulated in LMS, providing independent validation of our MS results (Fig. S1C). DES is characterised by elongated spindle-shaped cells set in a dense collagenous matrix^26^ which is in line with the enrichment of proteins involved in collagen organisation. No ontologies were enriched in SS but these tumours showed an upregulation of DNA damage response proteins particularly those involved in non-homologous end joining (NHEJ), including the catalytic subunit of DNA-dependent protein kinase (PRKDC), XRCC1, XRCC5, XRCC6, RAD50 and MRE11 (Fig. S1B and Supplementary Data 3), suggesting that exploiting double-strand break repair mechanisms could be an important therapeutic avenue in this histological subtype^27,28^. We further evaluated if pre-operative treatment impacts the enrichment of DNA damage response proteins in SS. Analysis of cases that had undergone pre-operative treatment or those that did not finds that these 6 proteins were similarly enriched in both groups of patients when compared to the rest of the STS cohort (Fig. S2 and Supplementary Data 5), indicating that the observed upregulation of DNA damage response proteins in SS is inherent in the biology of this subtype and not dependent on pre-treatment status. The ability of MS-based proteomics to capture known subtype-specific molecular processes in FFPE tissue specimens highlights the validity of this approach for biological discovery.

### Proteomic profiling of LMS identifies 3 molecular subtypes with distinct biological features and survival outcomes

LMS is one of the most common sarcomas subtypes accounting for ~20% of newly diagnosed STS^25^. It is typified by clinical heterogeneity in treatment responses, rates of metastasis and patient outcomes^11,29–31^. In keeping with these clinical observations, several studies have identified transcriptomic LMS subtypes with distinct clinicopathological features and biological pathways^12,32–35^. However, the presence of these molecular subtypes and the activation of these pathways remains to be verified at the protein level.

**Fig. 3:**
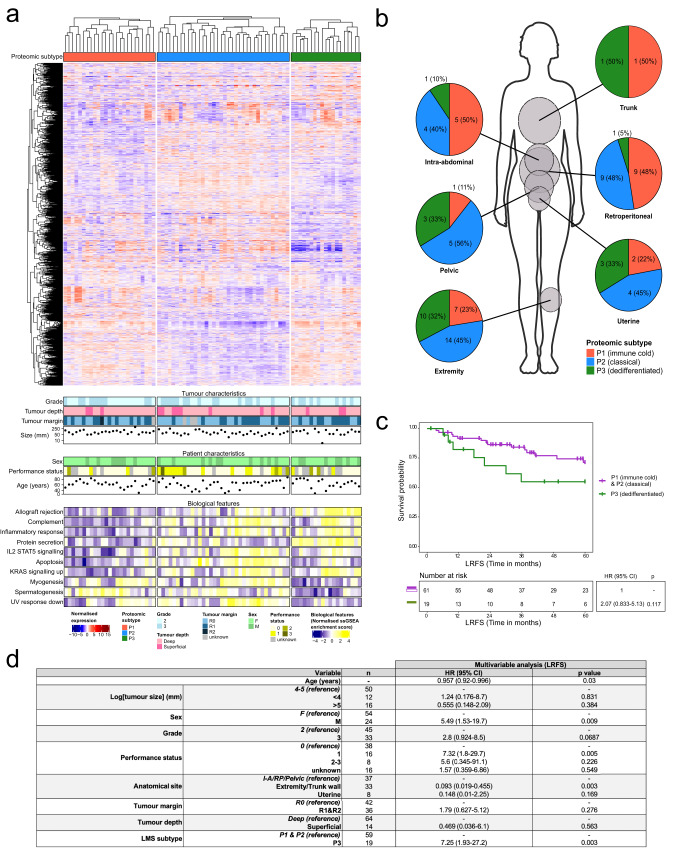
Leiomyosarcoma (LMS) is comprised of three proteomic subtypes. **a** Annotated heatmap showing the unsupervised clustering (Spearman distance) of 3262 proteins across LMS cases (*n* = 80), arranged by proteomic subtype (top annotation). Bottom annotations indicate key tumour and patient characteristics, and significant (one-way ANOVA; FDR < 0.001) biological features obtained from single sample Gene Set Enrichment Analysis (ssGSEA) of the MSigDB Hallmark gene sets (Supplemental Data 6A, B). **b** Pie charts depicting the breakdown of LMS proteomic subtypes at different anatomical sites. **c** Kaplan–Meier plot of local recurrence-free survival (LRFS) across the LMS proteomic subtypes stratified by P3 and P1/P2 combined. Hazard ratio (HR), 95% confidence intervals (CI) and *p*-value determined by univariable Cox regression. **d** Multivariable Cox regression assessing local recurrence-free survival (LRFS) in patients categorised by leiomyosarcoma (LMS) proteomic subtype. I-A intra-abdominal, RP retroperitoneal.

To determine whether proteomic data can account for the heterogeneity observed in LMS, consensus clustering of the proteomic dataset in our cohort of 80 LMS cases was performed (Fig. S3A–D). The baseline clinicopathological features of this LMS cohort are presented in Table S1. Three consensus proteome-based clusters (P1–P3) were identified which were determined to be significant by SigClust (*p* < 0.001) (Fig. 3a). Assessment of key tumour features (FNCLCC grade and tumour size, depth and margins) as well as patient characteristics (sex, performance status and age) showed no association with any of the three clusters identified (Fig. 3A and Table S1). By undertaking single sample gene set enrichment analysis (ssGSEA) for each case within the LMS cohort, we demonstrate that the proteomic clusters have distinct biological features. P1 is characterised by significantly lower ssGSEA scores for inflammatory response and KRAS signalling compared to P2 (*p* < 0.001) and P3 (*p* < 0.001) (Fig. 3a, Supplementary Data 6). In line with the observation of reduced inflammatory response, LMS cases in P1 displayed an “immune cold” phenotype with less CD3+ and CD4+ tumour infiltrating lymphocytes (TILs) compared to the other two clusters (*p* = 0.008) (Fig. S4A, B). However, CD8+ TILs were not different across the three clusters. P3 showed a marked reduction in ssGSEA scores for myogenesis compared to P1 (*p* = 0.001) and P2 (*p* < 0.001) (Fig. 3a, Supplementary Data 6). We further show that known smooth muscle protein markers including CFL2, SLMAP, MYLK, MYH11, and ACTA2 are significantly decreased in P3 versus P1 and P2 (Fig. S4C)^36^. A subset of cases in P3 segregated from the other LMS tumours in the UMAP analysis of the full STS cohort (Fig. S4D) which is in keeping with a “dedifferentiated” form of LMS that has previously been reported in transcriptomic and immunohistochemistry (IHC)-based studies^32,36^. Finally, P2 had relatively high levels of both myogenesis and inflammatory response ssGSEA scores and we termed this cluster the “classical” subtype. Significance analysis of microarrays (SAM) and prediction analysis of microarrays (PAM) was applied to the full dataset to identify a reduced subset of proteins that enables accurate classification of the three subtypes. This resulted in a reduced set of 153 proteins with an overall misclassification error rate of 0.037 for the three LMS proteomic subtypes (list of proteins provided in Supplementary Data 7).

Next, we determined the distribution of the three LMS proteomic subtypes by anatomical site. There was a clear dichotomy in subtype proportion with the retroperitoneal and intra-abdominal cases being enriched for the immune cold and classical subtypes with an underrepresentation of the dedifferentiated subtype; while the extremity, uterine and pelvic cases showed a different distribution with around half of the cases being classical, a third dedifferentiated and the remainder immune cold (Figs. 3b and S4E). We then evaluated the 5-year survival outcomes of patients in the three proteomic subtypes (Fig. 3c, d). In multivariable analysis, the dedifferentiated subtype had significantly inferior local relapse-free survival (LRFS) outcomes compared to the immune cold and classical (HR 7.25, 95% CI 1.93–27.2, *p* = 0.003) subtypes (Fig. 3d and Table S2). There was no difference in metastasis-free survival (MFS) and overall survival (OS) between the three proteomic subtypes (Fig. S4F). Taken together, our analyses provide evidence that LMS is comprised of three proteomic subtypes with distinct biological features, anatomical site distribution and LRFS outcomes.

### CD3+ TIL-low UPS and DDLPS harbour elevated levels of complement cascade proteins

**Fig. 4:**
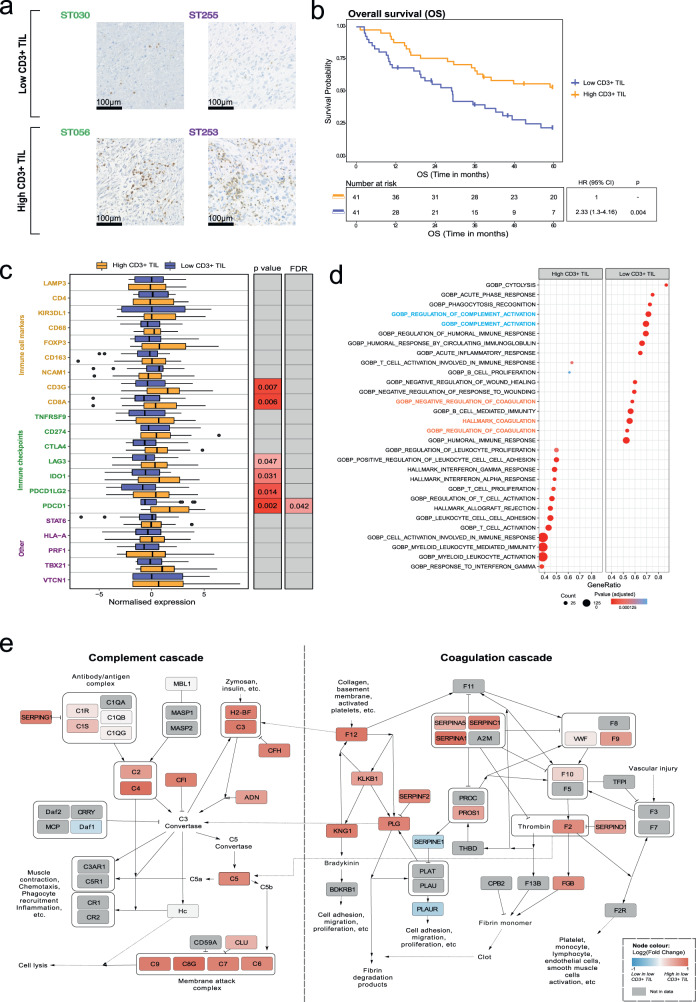
Characterisation of the immune profiles of dedifferentiated liposarcoma (DDLPS) and undifferentiated pleomorphic sarcoma (UPS). **a** Representative images of high and low CD3+ tumour infiltrating lymphocyte (TIL) staining from an exemplar in DDLPS (green) and UPS (purple) cases in the cohort. Samples were stratified as high and low based on median TIL counts (107 cells/mm^2^). **b** Kaplan–Meier plot of overall survival (OS) in CD3+ TIL-high and -low patients (*n* = 82). Hazard ratio (HR), 95% confidence intervals (CI) and *p*-value determined by univariable Cox regression. **c** Boxplots comparing expression of 21 immune-related genes in CD3+ TIL-high and -low cases. Boxplots indicate 25th, 50th, and 75th percentile, with whiskers extending from 25th percentile−(1.5*IQR) to 75th percentile+(1.5*IQR), and outliers plotted as points. *p* values determined by Kruskal–Wallis tests and adjusted to false discovery rate (FDR). **d** Gene set enrichment analysis (GSEA) results showing the top 15 gene sets enriched in CD3+ TIL-high and and-low cases based on normalised enrichment score (NES) with gene sets related to complement activity (blue) and coagulation processes (orange) highlighted. **e** To inspect the proteins contributing to the enrichment of complement and coagulation cascades in these tumours, protein-protein interaction (PPI) networks were constructed based on the Kyoto Encyclopaedia of Genes and Genomics (KEGG) and WikiPathways databases. Node colour indicates Log_2_(Fold Change CD3+ TIL low: CD3+ TIL high) protein expression. Grey indicates nodes that are not in the proteomic data. This analysis highlighted the serpin family of serine proteases to be strongly upregulated in low CD3+ TIL patients (SERPINA1/A5/C1/D1/F2/G1). Several complement proteins were also upregulated in low CD3+ TIL patients, including those of the membrane attack complex (MAC).

Data from clinical trials of anti-PD-1/PD-L1 immune checkpoint inhibitors (CPIs) have shown that a subset of UPS and DDLPS patients benefit from treatment with this class of drugs^37,38^. Additionally, correlative studies from the SARC028 trial indicate that responders to the anti-PD-1 antibody pembrolizumab harbour higher TIL densities compared to non-responders^39^. Here we sought to dissect the biological processes associated with TIL levels in these two subtypes. We first evaluated the levels of CD3+, CD4+, and CD8+ TILs in a subset of UPS (*n* = 50) and DDLPS (*n* = 32) patients for which there was sufficient tissue to generate tumour microarrays (Fig. 4a). The median TIL levels were as follows: CD3+ 107 cells/mm^2^ (range: 1–1239), CD4+ 89 cells/mm^2^ (range: 1–1735) and CD8+ 31 cells/mm^2^ (range: 0–869). Stratifying patients into CD3+ TIL-high or -low groups based on median TIL counts showed that the CD3+ TIL low cases had a significantly poorer OS in univariable (HR 2.33, 95% CI 1.3–4.16, *p* = 0.004) and multivariable (HR 2.07, 95% CI 1.01–4.23, *p* = 0.048) analysis (Fig. 4b, and Table S3). A similar trend was observed for LRFS (HR 2.04, 95% CI 1.03–4.04, *p* = 0.04) but not MFS (Fig. S5).

To evaluate if other immune cell markers and checkpoint molecules are associated with CD3+ TIL-high or -low subgroups, we undertook targeted gene expression analysis of 21 key immune genes (Supplementary Data 8). Several genes involved in immune checkpoint regulation were elevated in CD3+ TIL high versus low cases (Fig. 4c). These include *PDCD1* (which encodes for the PD-1 receptor), *PDCD1LG2* (which encodes for the PD-L2 ligand), *IDO1* and *LAG3*. Only *PDCD1* remained significant following multiple testing correction. Of the 21 immune genes analysed, 3 were present in the proteomics dataset (*CD163*, *NCAM1* and *STAT6*). Spearman’s rank correlation analysis of gene expression versus protein expression levels showed a poor correlation for *CD163* (*ρ* = 0.46, *p* < 0.001) and *STAT6* (*ρ* = 0.39, *p* = 0.001) and a moderate correlation for *NCAM1* (*ρ* = 0.59, *p* < 0.001).

Given that UPS and DDLPS patients with low CD3+ TIL levels are considered to have “immune cold” tumours and unlikely to benefit from anti-PD-1/PD-L1 inhibitors, we mined the proteomic data to establish if other biological pathways that could be exploited for therapy were present. Gene set enrichment analysis (GSEA) showed that CD3+ TIL-high patients had enrichment of ontologies associated with T-cell activation, T-cell receptor signalling, leucocyte proliferation and cell adhesion, and interferon responses (Fig. 4d). In contrast, patients in the CD3+ TIL-low subgroup were enriched for ontologies comprising the complement cascade and its closely related pathway, the coagulation cascade (Supplementary Data 9). These proteins include serine proteases in the two pathways that are thought to originate from the same ancestral genes^40^, the serpin family of serine protease inhibitors and components of the membrane attack complex (MAC) (Fig. 4e). These data indicate that while CD3+ TIL-low tumours have reduced levels of cellular immunity and expression of key immune checkpoint genes, these patients harbour an active complement system.

### Comparative analysis of transcriptomic and proteomic profiles of AS

AS is a rare and aggressive vascular subtype comprising <3% of all STS and arises from endothelial cells^41^. These cancers can be classified into two groups based on their aetiology. Primary AS arise de novo and develops primarily in younger patients (30–50 years of age) while secondary AS comprise radiation-associated or lymphedema-associated AS that present in older patients (median age of ~70 years)^42^. Here we performed RNA-seq on a subset of 25 AS cases in our cohort and undertook a comparative analysis of the transcriptomic and proteomic data.

**Fig. 5:**
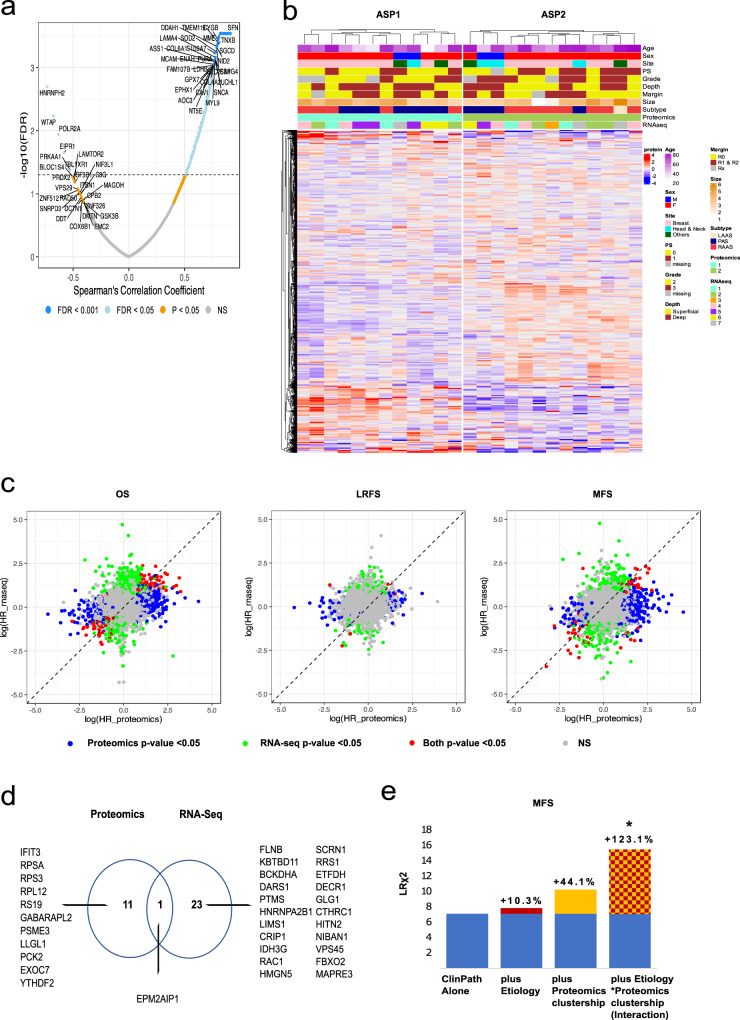
Comparative analysis of transcriptomic and proteomic profiles of angiosarcomas (AS). **a** Volcano plot showing Spearman’s correlation and -log10 transformed *p*-values for the 3383 genes/proteins. Negatively correlated genes/proteins with *p*-value < 0.005 and positively correlated genes/proteins with FDR < 0.001 are annotated on the plot. **b** Annotated heatmap of proteomic data (3383 proteins) for 25 AS cases. The samples were clustered using M3C method with *K*-means. From top to bottom, panels indicate age, sex, size, performance status, tumour grade, depth, margin, size and aetiology/subtype. The corresponding RNA-seq clusters (Fig. S7A) are shown. **c** Scatter plots of log2-transformed hazard ratios from univariate Cox regression models fitted using OS (left panel), LRFS (middle panel) and MFS (right panel) using gene/protein expression. Blue dots are proteins with a *p*-value < 0.05, green dots are genes with a *p*-value < 0.05 and red dots are the gene/proteins where both datasets returned a *p*-value < 0.05. **d** Venn diagram showing the overlap of the genes and proteins that are significantly associated with all the survival endpoint measures (OS, LRFS, and MFS). **e** Likelihood ratios (Chi-square) of the different Cox regression models and the relative improvement of prognostic information with the addition of different variables (aetiology, proteomics clustership or aetiology*proteomics clustership interaction) to models comprising only baseline clinicopathological variables alone. *p < 0.05. LAAS lymphedema-associated angiosarcoma, PAS primary angiosarcoma, RAAS radiation-associated angiosarcoma, PS performance status, Rx margin unknown.

We first assessed the correlation between the genes/proteins within the two datasets. Of the 3383 genes/proteins that were present in both datasets, 666 were significantly positively correlated (FDR < 0.05) with Spearman correlation coefficient *ρ* of 0.51–0.91 (Fig. 5a and Fig. S6). Several of the highly positively correlated genes/proteins include previously reported candidate sarcoma drug targets such as argininosuccinate synthetase 1 (ASS1), lactate dehydrogenase B (LDHB), melanoma cell adhesion molecule (MCAM)^43–45^. Interestingly, there were also 5 genes/proteins that were negatively correlated (*ρ* of −0.73 to −0.53, FDR < 0.05), including proteins involved in the regulation of RNA splicing (HNRPH2, WTAP, and POLR2A) (Fig. 5a).

To determine whether the use of proteomic or transcriptomic datasets can identify clinically meaningful expression patterns relating to the biology of AS, we performed Monte-Carlo consensus clustering (M3C) of the two datasets separately. This analysis identified 2 and 7 clusters for the proteomic and transcriptomic datasets respectively (Figs. 5b and S7A). Notably, the clusters of samples defined by the proteomic data were not further sub-classified by the clusterships defined by the RNA-seq data, suggesting that both datasets harboured distinct information about the biology of AS. To evaluate whether using the smaller number of proteins identified by MS had an impact on the clusterships defined by the transcriptomic data, we repeated the M3C using the full RNA-seq gene list of 9780 genes. There were only two samples assigned to a different membership of clusters when compared to the limited gene list (*n* = 3383), indicating that the reduction in the number of genes had minimal impact on the clustering results (Fig. S7B). Importantly, only the proteomic but not the RNA-seq data defined molecular clusters which were clinically meaningful: AS proteomic cluster 2 (ASP2) which is comprised mostly of secondary AS (radiation-associated and lymphedema-associated AS) and AS proteomic cluster 1 (ASP1) which is comprised of an almost equal mix of secondary and primary AS (Fig. 5b).

We sought to establish if biomarker(s) identified within each of the two datasets provide distinct prognostic information. Univariable Cox analysis was performed on the 3383 genes or proteins to determine the association and their significance with OS, LRFS or MFS (Fig. 5c). The scatter plots show that for each of the outcome measures assessed, there was a distinct set of genes or proteins that were prognostic for survival outcome (*p* < 0.05). The number of significant proteins (with HR > 2.0 or <0.5) is 439, 84, and 400 for OS, LRFS, and MFS, respectively, while the number of significant genes (with HR > 2.0 or <0.5) is 521, 115, and 375 for OS, LRFS, and MFS respectively. Of the 12 proteins and 24 genes that were significantly associated with all the survival endpoint measures (OS, LRFS, and MFS), only one protein/gene (EPM2AIP1) was overlapping (Fig. 5d). In addition, our analysis identified a subset of genes/proteins in which the gene and protein expression levels showed opposing associations with survival: OS (ROCK2, ALDH9A1, RTN1), LRFS (ZYX), and MFS (EDF1, PRSS1, CTSA, DDX5, NELFE, SARNP, SREK1, and MAT2B). Our analysis demonstrates the distinct and complementary nature of the proteomic and RNA-seq datasets in the identification of candidate prognostic factors for AS risk stratification.

We next used multivariable models to assess the additional prognostic information provided by the proteomic clusterships (ASP1 and ASP2) (Fig. 5b), RNA-seq clusterships (Fig. S7A), or aetiology (primary or secondary AS) compared to the use of baseline clinico-pathological variables (tumour grade, size and depth) alone (Fig. 5e). In univariable Cox regression analysis, the survival estimates of the RNA-seq clusterships could not be estimated as a result of extreme hazard ratios and infinite confidence intervals from the models and therefore was not included for multivariable analysis. Including the interaction between the proteomic clusterships and aetiology provided a gain of 123.1%, i.e. twice the prognostic information, compared to a model comprising of clinico-pathological variables only (MFS, change in LR*χ*² = 8.77). This model also outperformed the multivariable Cox models that added either aetiology or proteomic clusterships only. Collectively, these findings demonstrate the proteome-specific features that provide optimal risk classification for distant metastasis in AS.

### Sarcoma proteomic modules define pan-STS biological subgroups of prognostic value

**Fig. 6:**
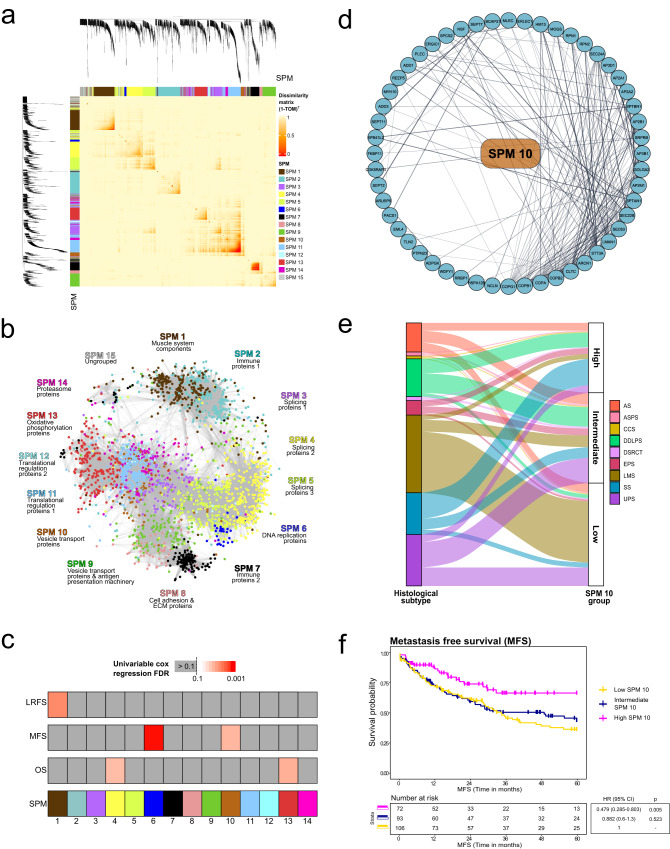
Sarcoma proteomic modules (SPM) are associated with patient survival outcomes. **a** Co-expression heatmap showing the correlation of protein expression based on topological overlap matrix (TOM) dissimilarity (1−TOM)^7^. Cluster dendrogram height indicates 1−Pearson’s correlation. **b** Protein co-expression network comprising 3290 nodes and 168,574 edges. Nodes indicate proteins and are coloured based on SPM membership. Edges show a correlation between protein expression, where a thicker line indicates a stronger correlation. Representative biological features are annotated for each module. **c** Overview of univariable Cox regression results for each SPM and local recurrence-free survival (LRFS), metastasis-free survival (MFS), and overall survival (OS). **d** Protein–protein interaction (PPI) network of SPM 10 comprising 94 nodes and 233 edges. Nodes are proteins and edges represent the StringDB database score between proteins, where a thicker line indicates a higher score (range = 0.401– 0.999). **e** Sankey diagram illustrating the distribution of histological subtype (excluding DES and RT) across three SPM10 subgroups. Subgroups identified by tertile stratification based on median SPM 10 expression across the full cohort. **f** Kaplan–Meier plot of MFS across the three SPM 10 subgroups. Hazard ratio (HR), 95% confidence intervals (CI) and *p*-value determined by univariable Cox regression.

We next established whether pan-sarcoma biological signatures defined by co-regulated proteins or protein complexes were intrinsic within the sarcoma proteomic dataset. Utilising weighted gene co-expression network analysis (WGCNA)^46^ on the dataset comprising proteins that were quantified across all samples (Figs. 6a and S8), we identified 14 distinct and 1 ungrouped Sarcoma proteomic modules (SPMs) comprising between 41 and 420 proteins (Supplementary Data 10). Constructing a protein co-expression network of 3290 nodes and 168,574 edges revealed SPMs comprising a broad range of biological functions including splicing, immunity, DNA replication, and cellular metabolism (Fig. 6b). We then evaluated the association of SPMs with LRFS, MFS and OS to identify prognostic biological signatures (Fig. 6c). For survival analysis, we removed RT as this is a paediatric disease and DES which unlike the other subtypes in the study is a locally infiltrative disease with no metastatic potential.

Two SPMs (SPM6 and SPM10) were associated with MFS (Fig. 6c). SPM6 has 41 proteins and is enriched in key components regulating DNA replication such as the minichromosome maintenance (MCM) complex as well as cell cycle proteins CDK1 and CDK2 (Fig. S9A). Analysis of the histological subtype breakdown of patients classified into SPM6-high, -intermediate and -low subgroups based on the median protein expression levels of the 41 proteins showed that this biological approach was subtype-agnostic with a broad representation of histotypes in each SPM6 subgroup (Fig. S9B). Patients in the SPM6-high subgroup had a significantly poorer MFS (HR 2.42, 95% CI 1.48–3.95, *p* < 0.001) compared to the SPM6-low (Fig. S9C) subgroup, which is in line with published studies showing that the MCM complex is a prognostic factor in multiple cancer types^47^. SPM10 has 94 proteins that comprise the intracellular vesicle transport machinery. These proteins include coatomer subunits (COPG1, COPA, COPB1/2, ARCN1), and components of the adaptor protein complexes (AP2M1, AP3B1, AP2B1, AP2A1/2, AP3D1) (Fig. 6d). Evaluation of the histological subtypes categorised as SPM10-high, -intermediate and -low subgroups showed that although there is some enrichment of histological subtypes in the different subgroups (e.g. LMS in the SPM10-low group), all histotypes were represented in the three SPM10 subgroups (Fig. 6e). In contrast to SPM6, patients in the SPM10-high subgroup had a significantly superior MFS compared to the SPM10-low subgroup (HR 0.479, 95% CI 0.285–0.803, *p* = 0.005) (Fig. 6F). Adjusting for clinicopathological factors including age, tumour size, grade, margins and depth, performance status and histological subtype, the SPM10 signature remained an independent prognostic factor (Table S4) in the multivariable Cox regression analysis. This analysis highlights the utility of a biological signature approach based on SPMs to refine clinical risk stratification for localised STS.

SAM and PAM were applied to identify a reduced subset of proteins that enables accurate classification of the three SPM10 subgroups. This resulted in a reduced set of 53 proteins with an overall misclassification error rate of 0.085 (a list of 53 proteins is provided in Supplementary Data 11). To assess if SPM10 has prognostic value beyond STS, we applied the centroids of this reduced set of proteins to the CPTAC breast cancer proteomic dataset^19^. We find that unlike STS, intermediate expression of SPM10 proteins in breast cancer is associated with poor OS and disease-specific survival (DSS) compared to SPM10-high and SPM10-low strata (log-rank OS: *p* = 0.003, DSS: *p* = 0.00057), suggesting that the utility of SPM10 as a prognosticator is likely to be cancer-type specific (Fig. S10).

## Discussion

This proteomic study of multiple sarcoma histological subtypes advances our current knowledge of the STS proteome which has thus far been restricted to low-resolution RPPA studies. By utilising an MS-based methodology that is compatible with standard FFPE specimens routinely collected for diagnostics, we were able to identify >8000 proteins with 3290 proteins quantified across all samples. We demonstrate the power of this approach to extend our understanding of STS biology and identify strategies for molecular-based disease classification, biomarker-informed prognostication and candidate therapeutic avenues.

LMS is a clinically and molecularly heterogenous disease and while several studies have reported transcriptomic-based molecular subgroups with defined biological pathways and clinical outcomes^12,32–35^, there are currently no consensus molecular definitions of LMS subtypes^48^. Here we show that at the protein level, LMS can be distinguished into three proteomic subtypes. The dedifferentiated proteomic subtype P3 is characterised by a reduction in smooth muscle protein expression and has inferior LRFS outcomes within our LMS cohort. Notably, our study shows that while retroperitoneal and intra-abdominal LMS cases have a much lower incidence of the dedifferentiated subtype, a third of uterine, pelvic and extremity cases comprise of this proteomic subtype, indicating that protein-based biomarkers could facilitate prognostication of LMS tumours from the same anatomical site. It has previously been shown that UPS-like poorly differentiated LMS cases with progressive loss of smooth muscle markers (as measured by IHC) have poorer outcomes independent of tumour morphology^36^. This feature was similarly reported in the transcriptomic subtype 1 identified in a genomic study by Anderson et al. and transcriptomic subtype II defined by Guo et al.^32,34,48^. Given the agreement between proteomic, IHC and transcriptomic analyses across multiple studies and cohorts, we believe that there is a strong rationale to support a consensus definition of a poorly differentiated molecular LMS subtype with the prospective evaluation of protein-based biomarkers to aid patient risk stratification in the clinic.

Clinical trials and real world-experience of anti-PD-1/PD-L1 CPI use in sarcomas have shown that a subset of UPS and DDLPS patients derive clinical benefits from this class of drugs^37,38,49–51^. Furthermore, UPS has consistently been found to have the highest TIL levels across multiple histological subtypes^52–54^. Candidate biomarkers of CPI response in sarcoma patients include TIL density, presence of tertiary lymphoid structures, PD-1/PD-L1 expression and transcriptomic-based sarcoma immune classes^39,55–57^. A common theme of these putative biomarkers involves the identification of the “immune hot” subset of patients who are more likely to respond to CPIs. An outstanding question remains as to the therapeutic options for the vast majority of STS patients who are classified as “immune cold” and therefore are not ideal candidates for CPI therapy. By mining the proteomic dataset in the CD3 + TIL-low subset of UPS and DDLPS patients, we show that these tumours are enriched in components of the complement-mediated innate immune response. Studies in other cancer types have reported that complement activation promotes tumour growth and suppresses anti-tumour immunity including levels of CD8+ and CD4+ TILs^58–61^ which is consistent with the inferior survival outcomes in the CD3+ TIL-low UPS and DDLPS patients in our cohort. In addition, combined blockade of PD-1/PD-L1 and complement proteins has been shown to restore antitumour immune responses with synergistic effects in lung and colon cancer murine models^58,62^. Very recently, Magrini et al. demonstrated that complement activation is immune suppressive and has a pro-tumoral role in sarcoma mouse models of UPS and in sarcoma patients. They further showed that preclinical inhibition of the complement pathway potentiates anti-PD-1 therapy^63^. Complement antagonists such as the anti-C5 monoclonal antibody eculizumab which is FDA-approved for paroxysmal nocturnal haemoglobinuria and other antagonists including pexelizumab, TP-10, MLN-2222 which are in advanced clinical development for coronary artery bypass grafting, represent opportunities for cancer therapy repurposing^64^. Our data, therefore, provide support for future preclinical and clinical evaluation of complement inhibitors in CD3+ TIL-low UPS and DDLPS with the potential for combination therapy with anti-PD-1/PD-L1 CPIs.

Several large-scale comparative studies have shown that the correlation of protein and mRNA levels in cells and tissues is generally poor^65–67^. Reasons for the poor global correlation between mRNA and protein levels are multi-factorial and may include differences in mRNA and protein abundance as well as turnover^68,69^. By undertaking a comparative analysis of proteomic and transcriptomic data, we demonstrate that only ~20% of proteins in our dataset are significantly correlated with mRNA levels in AS. Consistent with a previous study of 9 human cell lines and 11 human tissues^65^, we find that ASS1 is highly correlated at both the mRNA and protein levels. Loss of ASS1 confers a synthetic lethal interaction to arginine deprivation (with pegylated arginine deiminase) and chloroquine combination therapy in sarcomas^43^. Our data suggest that both ASS1 protein and mRNA levels may be used as a biomarker for this therapeutic strategy. Furthermore, we show that proteomic but not transcriptomic data identified two molecular subgroups (ASP1 and ASP2) that were clinically meaningful in separating AS subtypes with distinct aetiology. Our analysis also indicates that mRNA and proteins provide distinct and complementary prognostic information and few genes/proteins can be used interchangeably as prognostic clinical biomarkers in AS. Notably, only the incorporation of proteomics data into a multivariable Cox model led to a significantly increased (nearly two-fold) improvement in prognostic information compared to a model comprising only baseline clinicopathological variables. Given the relatively small patient numbers in our cohort, these promising results need to be independently validated in future studies.

We defined 14 SPMs which are biological signatures that capture a broad spectrum of STS functional biology. To demonstrate the proof-of-principle utility of these signatures, we identified several SPMs which are associated with survival. We further show that when categorised by high, intermediate or low subgroups, these tumours share protein-based molecular characteristics that transcend histological subtype. Current clinical management of STS in the localised setting, including risk stratification and treatment selection, is largely reliant on histological subtype, anatomical site and other factors including size and grade^4^. Conceptually, our findings indicate that in addition to current histotype-focused strategies, an orthogonal and complementary biological signature-driven approach may also aid clinical decision-making. Several gene expression prognostic signatures based on a priori-defined biological pathways such as chromosome integrity and hypoxia have been reported^13,70,71^. However, our study is distinct in that the SPMs were derived de novo from the sarcoma proteomic dataset with no prior knowledge of specific biological pathways. The power of this discovery-based approach is demonstrated by the finding that a vesicle transport protein signature (SPM10) is an independent positive prognostic indicator for MFS. Previous reports have found that gene expression levels of coatomer components *COPB1* and *COPB2* are not associated with survival in the TCGA SARC dataset^72,73^. However, recent studies have shown that a deficiency in *COPA* or *COPG1* leads to deregulation of the immune system resulting in immunodeficiency^74,75^. Since sarcoma patients with immune cold tumours have poorer outcomes compared to those with immune hot tumours^56^, one hypothesis is that sarcoma patients with low SPM10 protein expression levels have poor immune cell function and therefore inferior MFS outcomes. This hypothesis needs to be functionally tested in future experiments. The use of SPMs could have a clinical impact in improving sarcoma cure rates by identifying high-risk patients that may benefit from intensified treatment regimens including peri-operative chemotherapy. Our approach paves the way for future studies that combine SPMs with established nomograms such as Sarculator and PERSARC to develop integrated tools for improved risk classification of STS patients^76–78^.

There are several limitations to our study. Our analysis was performed on a retrospective cohort which is susceptible to selection bias. Our findings should thus be considered hypothesis-generating and require future validation in independent cohorts. In addition, several sarcoma subtypes are known to harbour extensive intra-tumoural heterogeneity^79,80^ and our bulk proteomic approach is unable to resolve the individual contribution of distinct heterogenous tumour regions to the aggregate proteomic data. Despite this limitation, we are able to readily identify both previously reported and new findings associated with STS biology, highlighting the utility and validity of our approach. Future studies incorporating emerging spatial and single-cell proteomic technologies could shed light on the impact of intra-tumoural heterogeneity on protein-based signatures. However, unlike MS-based bulk proteomics, the cost of deploying such methodologies in the routine clinical setting is prohibitive and will therefore likely remain research use-only tools. Finally, our study has focused on localised disease and given the clonal evolution of tumours that have recently been reported in several sarcomas subtypes^32,81,82^, it remains to be determined if our findings will apply to locally relapsed and metastatic tumours.

In conclusion, we have developed a valuable proteomic resource for the sarcoma community which is rich in biological and linked long-term clinical data. While the reduced set of proteins identified for LMS subgroup classification as well as SPM10 prognostication is relatively large and can only be evaluated by MS as opposed to conventional IHC, advances in targeted MS assays such as multiple/selective reaction monitoring (MRM/SRM) means that such analyses can be done within clinically meaningful timescales, as recently demonstrated by the use of this strategy in COVID-19 vaccine trials^83^. We anticipate that this proteomic resource will facilitate the discovery of pathophysiological mechanisms, new therapeutic strategies and candidate biomarkers to catalyse future advances in basic and translational sarcoma research.

## Methods

### Patient cohort selection

This research complies with all relevant ethical regulations. Retrospective collection and analysis of formalin-fixed paraffin-embedded (FFPE) tissue and associated clinical data were approved as part of the Royal Marsden Hospital (RMH) PROgnoStic and PrEdiCTive ImmUnoprofiling of Sarcomas (PROSPECTUS) study (NHS Research Ethics Committee Reference 16/EE/0213), National Taiwan University Hospital (Research Ethics Committee Reference 201912226RINB), and Newcastle University as part of Children’s Cancer and Leukaemia Group (CCLG) Biological Study 2012 BS 05 (Research Ethics Committee Reference 8/EM/0134). Written informed consent was obtained from participants. Patients were selected for inclusion based on the availability of sufficient primary tumour tissue in institutional archives from the three institutions. Diagnoses were confirmed by an expert histopathological review by soft tissue pathologists (K.T., C.F.). Baseline clinicopathological characteristics and survival data were collected by retrospective review of medical records.

Each FFPE block underwent histologic assessment through a review of haematoxylin and eosin (H&E) stained sections. Cases with >75% tumour cell content were subjected to downstream sample preparation workflows while those cases with <75% tumour cells were macrodissected to enrich for tumour content prior to sample preparation^84^. Liposarcoma (LPS) samples were assessed for well-differentiated (WD) and de-differentiated (DD) areas and macrodissected to enrich for regions with DD histology. Of a total of 390 samples that were identified from our archive, 41 samples were dropped due to poor protein extraction yields following lysis, 12 samples dropped due to poor peptide yields following protein digestion and 16 samples dropped due to poor proteome coverage following MS data acquisition, resulting in a final cohort of 321 cases (Fig. S11).

### Proteomics analysis

#### Protein extraction and digestion

Each tumour sample was deparaffinised with 3 xylene washes, rehydrated twice in a decreasing ethanol gradient (100%, 96%, 70%), and dried in a SpeedVac concentrator (Thermo Scientific, Waltham, MA, USA). Lysis buffer (0.1 M Tris–HCl pH 8.8, 0.5% (w/v) sodium deoxycholate, 0.35% (w/v) sodium lauryl sulfate) was added at 200 μL/mg of dried tissue, samples homogenised by 3 × 30 s pulses with a LabGen700 blender (ColePalmer, Vernon Hills, IL, USA), sonicated on ice for 10 min, and heated to 95 °C for 1 h to reverse formalin crosslinks. Lysis was performed for 2 h by shaking at 750 rpm at 80 °C. Samples were centrifuged at 14,000 × *g* at 4 °C for 15 min, the supernatant retained, and protein concentration measured by bicinchoninic acid (BCA) assay (Thermo Scientific Pierce, Waltham, MA, USA). Tissue extracts were digested by filter-aided sample preparation (FASP), as previously described^85^. Briefly, samples were concentrated in Amicon-Ultra 4 centrifugal filter units (Merck Group, Darmstadt, Germany), and detergents were removed by washing with 8 M urea. Samples were transferred to Amicon-Ulta 0.5 filters (Merck Group, Darmstadt, Germany), reduced with 10 mM dithiothreitol (DTT) for 1 h at 56 °C, and alkylated with 55 mM iodoacetamide (IAA) for 45 min at room temperature in the dark. Samples were washed with 100 mM ammonium bicarbonate (ABC) and digested with trypsin (Promega, Madison, WI, USA) at a ratio of 1:100 µg sample at 37 °C overnight. Peptides were collected by three centrifugations at 14,000 × *g* with 100 mM ABC, desalted using SepPak C18 Plus cartridges (Waters, Milford, MA, USA), and dried in a SpeedVac concentrator (Thermo Fisher Scientific, Waltham, MA, USA).

#### Tandem-Mass-Tag labelling

Dried peptides were labelled with TMT 11-Plex reagents (Thermo Scientific, Waltham, MA, USA) as per the manufacturer’s guidelines. For the 11th (131C) channel, a pooled reference containing lysates from LMS, DDLPS, UPS, and SS cases was used in all MS experiments. Briefly, samples were incubated with respective TMT labels for 1 h at room temperature, and the reaction was quenched with 5% hydroxylamine. Labelled peptides were pooled, dried in a SpeedVac concentrator, and desalted with SepPak C18 Plus cartridges as before.

#### High-pH reverse-phase fractionation

All samples were fractionated offline by Dionex UltiMate3000 HPLC system (Thermo Fisher Scientific, Waltham, MA, USA). Each sample was dissolved in 100 µL of solvent A (0.1% NH_4_OH in water), sonicated for 5 min and centrifuged at 15,000 × *g* for 2 min. Supernatant was loaded onto a 2.1 × 150 mm, 5 µm Waters (Milford, MA, USA) XBridge C18 column (5 µm particles) at a flowrate of 200 µL/min and peptides were separated using a gradient of 5–40% of solvent B (0.1% NH_4_OH in acetonitrile) for 30 min followed by 40–80% of solvent B in 5 min and held at 80% for additional 5 min. Overall 90 fractions (30 s per fraction) were collected by an automatic fraction collector into a 96 well-plate and combined into 10 fractions with a stepwise concatenation strategy. Pooled fractions were dried in SpeedVac concentrator.

#### Liquid chromatography and mass spectrometry

The liquid chromatography (LC)/MS analysis was performed on a Dionex UltiMate3000 HPLC coupled with the Orbitrap Fusion Lumos Mass Spectrometer (Thermo Scientific, Waltham, MA, USA). Each peptide fraction was dissolved in 40 μL of 0.1% formic acid and 10 μL were loaded to the Acclaim PepMap 100, 100 μm × 2 cm C18, 5 μm, trapping column (Thermo Fisher Scientific, Waltham, MA, USA) with a flow rate 10 μL/min. Peptides were then separated with the EASY-Spray C18 capillary column (75 μm × 50 cm, 2 μm) at 45 °C. Mobile phase A was 0.1% formic acid and mobile phase B was 80% acetonitrile, 0.1% formic acid. The gradient method at a flow rate of 300 nL/min included the following steps: for 120 min gradient from 5 to 38% B, for 10 min up to 95% B, for 5 min isocratic at 95% B, re-equilibration to 5% B in 5 min, for 10 min isocratic at 5% B. The precursor ions were selected at 120k mass resolution, with automatic gain control 4 × 10^5^ and ion trap for 50 ms for collision-induced dissociation (CID) fragmentation with isolation width 0.7 Th and collision energy at 35% in the top speed mode (3 sec). Quantification spectra were obtained at the MS3 level with higher-energy C-trap dissociation (HCD) fragmentation of the top 5 most abundant CID fragments isolated with Synchronous Precursor Selection (SPS) with quadrupole isolation width 0.7 Th, collision energy 65% and 50k resolution. Targeted precursors were dynamically excluded for further isolation and activation for 45 sec.

#### MS data processing

The SequestHT search engine in Proteome Discoverer 2.2 or 2.3 (Thermo Scientific, Waltham, MA, USA) was used to search the raw mass spectra against reviewed UniProt human protein entries (v2018_07 or later) for protein identification and quantification. The precursor mass tolerance was set at 20 ppm and the fragment ion mass tolerance was 0.02 Da. Spectra were searched for fully tryptic peptides with maximum 2 missed cleavages. TMT6plex at N-terminus/lysine and Carbamidomethyl at cysteine were selected as static modifications. Dynamic modifications were the oxidation of methionine and deamidation of asparagine/glutamine. Peptide confidence was estimated with the Percolator node. Peptide false discovery rate (FDR) was set at 0.01 and validation was based on *q*-value and decoy database search. The reporter ion quantifier node included an integration window tolerance of 15 ppm and an integration method based on the most confident centroid peak at the MS3 level. Only unique peptides were used for quantification, considering protein groups for peptide uniqueness. Peptides with average reporter signal-to-noise >3 were used for protein quantification. Proteins with an FDR < 0.01 and a minimum of two peptides were used for downstream analyses.

#### Proteomic data imputation and normalisation

All data were processed using custom R scripts in R v3.5.1 or later. Proteins identified in <75% of samples were removed, and those remaining were imputed using the *k*-nearest neighbour (*k*-NN) algorithm^86^. Data was normalised and batch effects were removed in a multi-step procedure. Firstly, each sample was divided by the corresponding reference sample, data was then log_2_ transformed, median-centred across samples, and standardised within samples. For subtype-specific analyses, data were first filtered for samples of interest, and protein filtering, imputation, and normalisation were performed as before.

### Immunohistochemistry (IHC)

Fourteen tissue microarrays (TMA) containing 63 LMS, 50 UPS and 32 DDLPS with at least 2 replicate cores were used for IHC. Consecutive 4 μm TMA sections were stained for H&E, CD3, CD4, and CD8 using the DAKO link automated stainer (Agilent, CA, USA). Sections were deparaffinised by xylene and rehydrated by graded ethanol. Antigen retrieval was performed using a DAKO FlexEnvision kit (K8002; Agilent, CA, USA) by either pressure cooking in citrate (pH 6) for 2 min (CD3) or incubating with pH 9 pre-treatment module (PTM) buffer (Agilent, CA, USA) for 20 min at 97 °C (CD4 and CD8). Incubation with primary antibody (CD3 DAKO M0452 at 1:600 dilution; CD4 DAKO 4B12 at 1:80 dilution; CD8 DAKO C8/144B at 1:100 dilution) was for 60 min at room temperature. Secondary antibody staining and visualisation were performed using DAKO FlexEnvision (Mouse) Kit, followed by the application of DAB and haematoxylin counterstaining. H&E slides were assessed to confirm viable tumour content, and CD3/4/8 + TIL stains were counted under direct brightfield microscopy at x400 magnification. For cores with section preservation of 50–100%, cell counts were corrected to 100% area. Data from cases where section preservation was <50% were excluded. Replicate scores were averaged and then multiplied by 1.274 to produce average CD3+, CD4+ or CD8+ TIL/mm^2^. Digital microscopy images for all stained TMA sections were captured at ×40 resolution using Nanozoomer-XR (Hamamatsu Photonics, Japan).

### NanoString gene expression analysis

Tumour total RNA was extracted using the All Prep DNA/RNA FFPE kit (Qiagen, Hilden, Germany) following the vendor’s standard protocol. mRNA concentrations were measured using Qubit fluorometric quantitation (Thermo Fisher Scientific, Waltham, MA, USA). RNA Integrity Number was measured using 2100 Bioanalyzer system (Agilent, CA, USA). RNA samples were stored at −80 °C until use. Targeted gene expression profiling was performed using a custom panel of 21 immune-related genes and 3 housekeeper genes with the nCounter PlexSet-96 platform (NanoString Technologies, Seattle, WA, USA). Total RNA of 150–450 ng (variable to account for RNA degradation) of tumour samples and calibration samples was input for hybridisation and analysis performed per manufacturer’s instructions using the nCounter Max system (NanoString Technologies, Seattle, WA, USA). The expression values of calibration samples were used to adjust for differences between PlexSet plates (i.e. technical variance). The calibrated raw expression data were then normalised using the NanoStringNorm R package by ‘CodeCount’ = ‘geo.mean’, ‘Background’ = ‘mean’, and ‘SampleContent’ = ‘housekeeping.geo.mean’. Additionally, values < 1 were set to 1, data log_2_ transformed and gene-level median centring was performed.

### Statistical methods

All statistical tests were two-sided and where required, *p* values were adjusted to false discovery rate (FDR) using the Benjamini–Hochberg procedure to account for multiple comparisons^87^. Where appropriate, the distribution of the data was assessed using Shapiro–Wilk tests for normality, and tests not assuming a normal distribution implemented if *p* < 0.05. Kruskal–Wallis one-way analysis of variance (ANOVA) tests, one-way ANOVA tests, Tukey’s honestly significant difference (HSD) tests, and chi-square tests of independence were implemented. Further details of specific statistical tests are listed in figure legends. Unless otherwise specified, data were analysed using custom R scripts in R v3.5.1 or later.

#### Clustering

To visualise the STS proteomic dataset, hierarchical clustering using Pearson correlation distance and dimension reduction by uniform manifold approximation and projection (UMAP)^88^ were used. To identify LMS molecular subtypes, consensus clustering (CC) was performed by agglomerative hierarchical clustering using Spearman’s rank with average linkage (ConsensusClusterPlus R package^89^). Protein and item (sample) resampling was set at 80% and CC was run for 1000 iterations for up to 10 clusters (*k*). Optimal *k* was determined through inspection of consensus matrices, the cluster tracking plot, the consensus cumulative distribution function (CDF) plot, the delta (Δ) area plot, and by calculating silhouette scores. Clusters were confirmed as statistically significantly different by SigClust with hard thresholding and 1000 sample simulations (*p* < 0.05)^90^.

#### Differential expression analysis

To identify upregulated proteins in histological subtypes with *n* > 20, 2-class unpaired significance analysis of microarrays (SAM) tests were performed using Student’s *t*-tests with an FDR < 1% and fold change ≥1.5 (samr R package, https://cran.r-project.org/web/packages/samr/samr.pdf).

#### Overrepresentation analysis (OA), gene set enrichment analysis (GSEA) and single sample GSEA (ssGSEA)

OA and GSEA were performed with ClusterProfiler in R^91^ using the gene ontology (GO) biological process (BP) and hallmark gene sets with between 9 and 501 genes^92,93^. Proteins were ordered by Log_2_-fold change, and for OA were filtered to those identified as uniquely upregulated in histological subtype by differential expression analysis. ssGSEA was performed using ssGSEA (v10.0.11) on the GenePattern public server^94^. Rank normalisation and a weighting exponent of 0.75 were used to assess enrichment of the hallmark gene sets containing at least 10 genes, and normalised enrichment scores were *z*-scored across gene sets. All gene sets were downloaded from the Molecular Signatures Database v7.5.1 and filtered for proteins within the proteomic dataset.

#### RPPA analysis

The level 4 (log2 transformed with loading and batch corrected) RPPA dataset from the TCGA-SARC study was downloaded from The Cancer Proteome Atlas portal (https://tcpaportal.org/tcpa/) and clinical data downloaded from the TCGA Pan-cancer Clinical Data Resource (TCGA-CDR) within the NCI Genomic Data Commons (https://gdc.cancer.gov/about-data/publications/PanCan-Clinical-2018). The RPPA dataset was feature level (protein) median centred across samples and plotted along with the TMT-MS data using box-and-whisker plots.

#### Weighted gene correlation network analysis (WGCNA)

WGCNA was performed using the WGCNA R package^46^. Normalised proteomic data was used to construct a co-expression network. Network type was specified as signed hybrid and constructed with an optimal soft threshold value (*β*) of 5, determined by graphical inspection of network scale-free topology and mean connectivity across a range of *β* values. Average linkage hierarchical clustering with dynamic cutting and a deep split of 2 was used to identify modules of ≥30 proteins, and 1−Pearson correlation cut height ≥ 0.25.

#### Protein–protein interaction (PPI) networks

All PPI networks were built in Cytoscape v3.9.1^95^. To assess the complement and coagulation cascades, WikiPathway WP558 (63 nodes) was imported, adapted to include the C5 axis, and layout manually applied. To visualise the SPM landscape, a protein co-occurrence matrix was used, with co-occurrence scores between pairs restricted to >0.05 and an edge-weighted spring-embedded layout used. To inspect individual SPM networks, the STRING database v11.0 was queried^96^, a confidence cut-off score of 0.4 was applied and a circular layout was used.

#### Survival analyses

The association of biomarker(s) with survival outcome were evaluated based on Kaplan–Meier survival estimates and multivariable Cox regression analyses adjusted for standard clinicopathological variables. Tumour size showed non-linearity in relation to outcome, therefore the variable was log-transformed and martingale residuals were used to identify optimal cutpoints for categorisation. The three survival outcome endpoints (events) are as follows: (1) local recurrence-free survival (LRFS) defined as the time from primary disease surgery to radiologically confirmed local recurrence or death, (2) metastasis-free survival (MFS) defined as the time from primary disease surgery to radiologically confirmed metastatic disease or death, (3) overall survival (OS) defined as the time from primary disease surgery to death from any cause. Patients who do not have events were censored at their last follow-up time, up to 5 years. The significance of differential survival was evaluated by Wald tests.

#### SAM–PAM analysis

Significance analysis of microarrays (SAM) and prediction analysis of microarrays (PAM) were performed to reduce the list of proteins for the SPM10 and LMS subgroups. siggenes (https://www.bioconductor.org/packages/release/bioc/html/siggenes.html) and pamr (https://tibshirani.su.domains/PAM/Rdist/doc/readme.html) packages were used for performing SAM and PAM, respectively. SPM10 consists of 94 proteins expression in a total of 271 samples. LMS consists of a total of 3262 proteins expression in 80 samples. *Z*-score was applied to protein expression data before performing SAM. SAM analysis was performed with high, inter and low group labels (for the SPM10 dataset) and P1–P3 group labels (for the LMS dataset). This analysis gave a set of proteins with a set of delta values for each dataset. PAM analysis with 10-fold cross-validation was then performed on this protein set. A final protein set for each dataset was chosen based on minimum overall misclassification error. For SPM10, the PAM centroids obtained for the selected protein set were then used to predict high, inter and low subtypes of CPTAC breast cancer samples^19^. Survival analysis was also performed on CPTAC breast cancer samples with the subtypes obtained. Log-rank test *p*-value < 0.05 was set as the level of significance.

#### Comparative analysis of the angiosarcoma cohort

Details of the comparative transcriptomic and proteomic analysis of the angiosarcoma cohort are provided in the Supplementary Methods.

### Reporting summary

Further information on research design is available in the Nature Portfolio Reporting Summary linked to this article.

## Supplementary information

Supplementary information

Description of Additional Supplementary Files

Supplementary Data 1

Supplementary Data 2

Supplementary Data 3

Supplementary Data 4

Supplementary Data 5

Supplementary Data 6

Supplementary Data 7

Supplementary Data 8

Supplementary Data 9

Supplementary Data 10

Supplementary Data 11

Reporting Summary

## Data Availability

The raw proteomic data generated in this study have been deposited in the ProteomeXchange Consortium via the PRIDE partner repository^97,98^ with the dataset identifier PXD036226. The raw transcriptomic data are deposited at the European Genome-phenome Archive (EGA)^99^, which is hosted by the EBI and the CRG, under accession number EGAD00001010839. To protect patient privacy, as required by law, access to the raw transcriptomic data deposited in the EGA is controlled by the Data Access Committee (DAC) of the Institute of Cancer Research. All researchers can obtain access by submitting a project proposal to the DAC by contacting the corresponding author (P.H.H.). Requests will be handled within ~2 weeks. The DAC will also determine the length of permitted access. The clinical data is available under restricted access due to data privacy legislation, access can be obtained by contacting the corresponding author (P.H.H.) and will require the researcher to sign a data access agreement with the Institute of Cancer Research after approval by the DAC. The DAC will determine the length of permitted access with an expected response timeframe of ~2 weeks for access requests. The normalised proteomic dataset and normalised NanoString dataset are provided in the Supplementary Information. The TCGA SARC RPPA data is available from The Cancer Proteome Atlas portal (https://tcpaportal.org/tcpa/) and clinical data are available from the TCGA Pan-cancer Clinical Data Resource (TCGA-CDR) within the NCI Genomic Data Commons (https://gdc.cancer.gov/about-data/publications/PanCan-Clinical-2018). The raw mass spectra were searched against UniProt human protein entries (v2018_07 or later) for protein identification and quantification (https://www.uniprot.org/proteomes/UP000005640). Source data are provided with this paper.

## Source data

Source data file
