# Supplementary material for: The proteomic landscape of soft tissue sarcomas
Source: Nat Commun. 2023 Jun 29;14:3834. doi: 10.1038/s41467-023-39486-2 (PMC10310735; doi:10.1038/s41467-023-39486-2)
Supplement: Supplementary file 1 — Supplementary information [file 41467_2023_39486_MOESM1_ESM.pdf]

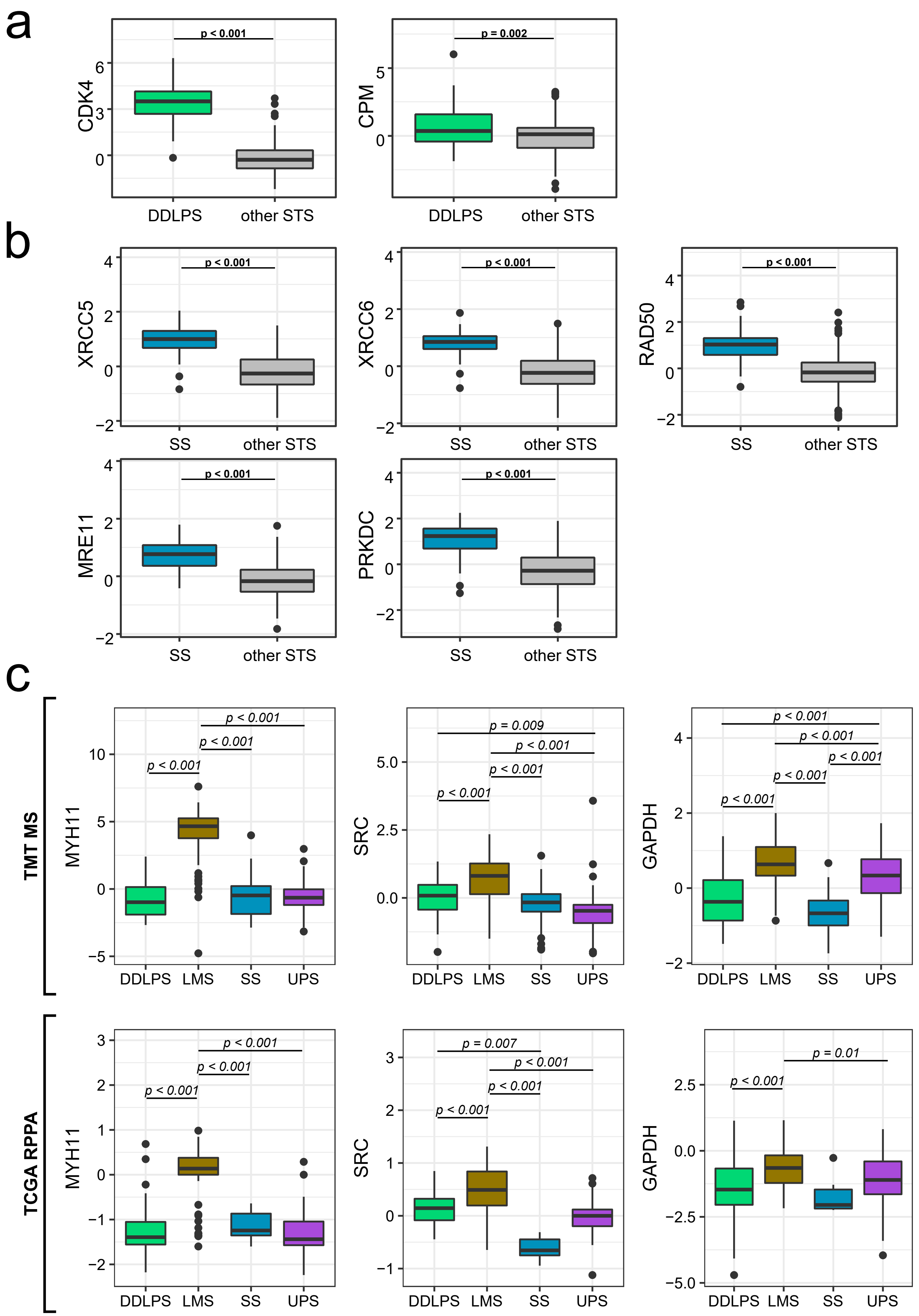

a
CDK4
p < 0.001
DDLPS
other STS
CPM
p = 0.002
b
XRCC5
XRCC6
RAD50
MRE11
PRKDC
SS
other STS
c
TMT MS
TCGA RPPA
MYH11
SRC
GAPDH
p < 0.001
p = 0.009
p = 0.007
p = 0.01
DDLPS
LMS
SS
UPS

Supplementary Figure 1. Subtype-enriched protein abundance. (a-b) Boxplots showing the normalised abundance of a subset of proteins identified as uniquely upregulated in dedifferentiated liposarcoma (DDLPS;**a**) and synovial sarcoma (SS;**b**) compared to other soft tissue sarcoma (STS) subtypes (FDR<1%, foldchange ≥ 1.5 as per significance analysis of microarray analysis in Supplementary Data 3).Boxplots indicate 25th (minima), 50th (centre), and 75th percentile (maxima), with whiskers extending from 25th percentile-(1.5*IQR), to 75th percentile+(1.5*IQR), and outliers plotted as points. DDLPS (n=39, STS n=282), SS(n=43, STS n=278). **(c)** Boxplots showing the normalised abundance of proteins uniquely upregulated in leiomyosarcoma (LMS) compared to DDLPS , SS and undifferentiated pleomorphic sarcoma (UPS) in two independent cohorts. Top panel shows normalised protein abundance based on tandem mass tag (TMT) mass spectrometry (MS) data from our cohort (DDLPS n=39, LMS n=80, SS n=43, UPS n=53). Bottom panel shows normalised protein abundance based on the reverse-phase protein array (RPPA) data from The Cancer Genome Atlas (TCGA) Sarcoma cohort (DDLPS n=50, LMS n=80, SS n=10, UPS n=44). Boxplots indicate 25th (minima), 50th (centre), and 75th percentile (maxima), with whiskers extending from 25th percentile-(1.5*IQR), to 75th percentile+(1.5*IQR), and outliers plotted as points. Significance determined by one-way analysis of variance (ANOVA) followed by Tukey's honesty significant difference (HSD) tests. Source data are provided as a Source Data file.

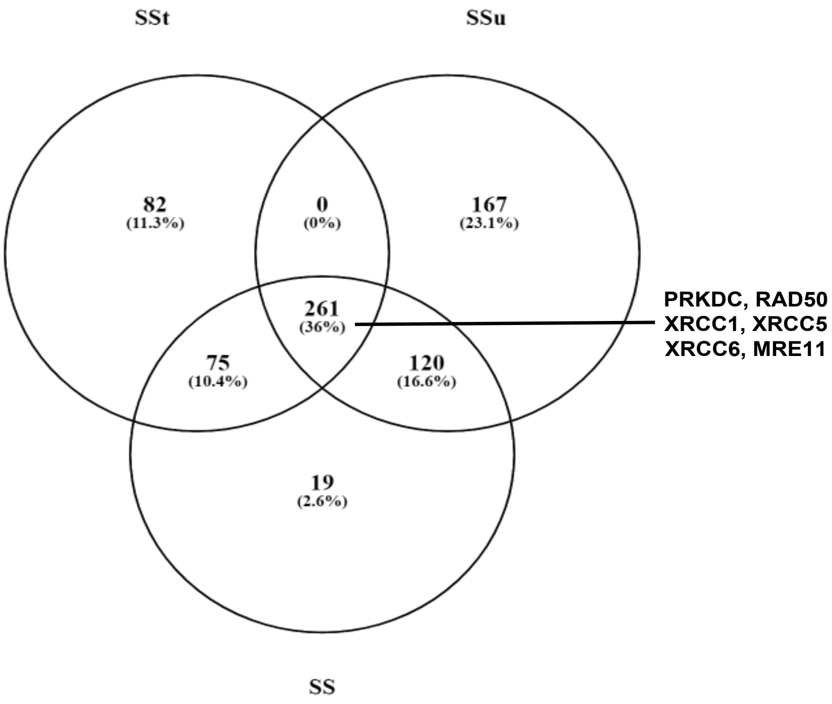

Supplementary Figure 2. Comparison of significantly enriched proteins in synovial sarcoma
Venn diagram indicating the overlap of significantly upregulated proteins in 1. all synovial sarcoma cases in the cohort (SS), 2. the subset of cases that underwent neoadjuvant treatment(SSt) and 3. the subset of cases that did not receive neoadjuvant treatment (SSu). Notably, the six DNA repair proteins (PRKDC,RAD50, XRCC1, XRCC5, XRCC6 and MRE11) are found in all three groups indicatingtheenrichmentofDNA repair proteins is not treatment related but intrinsic to the synovial sarcoma subtype.

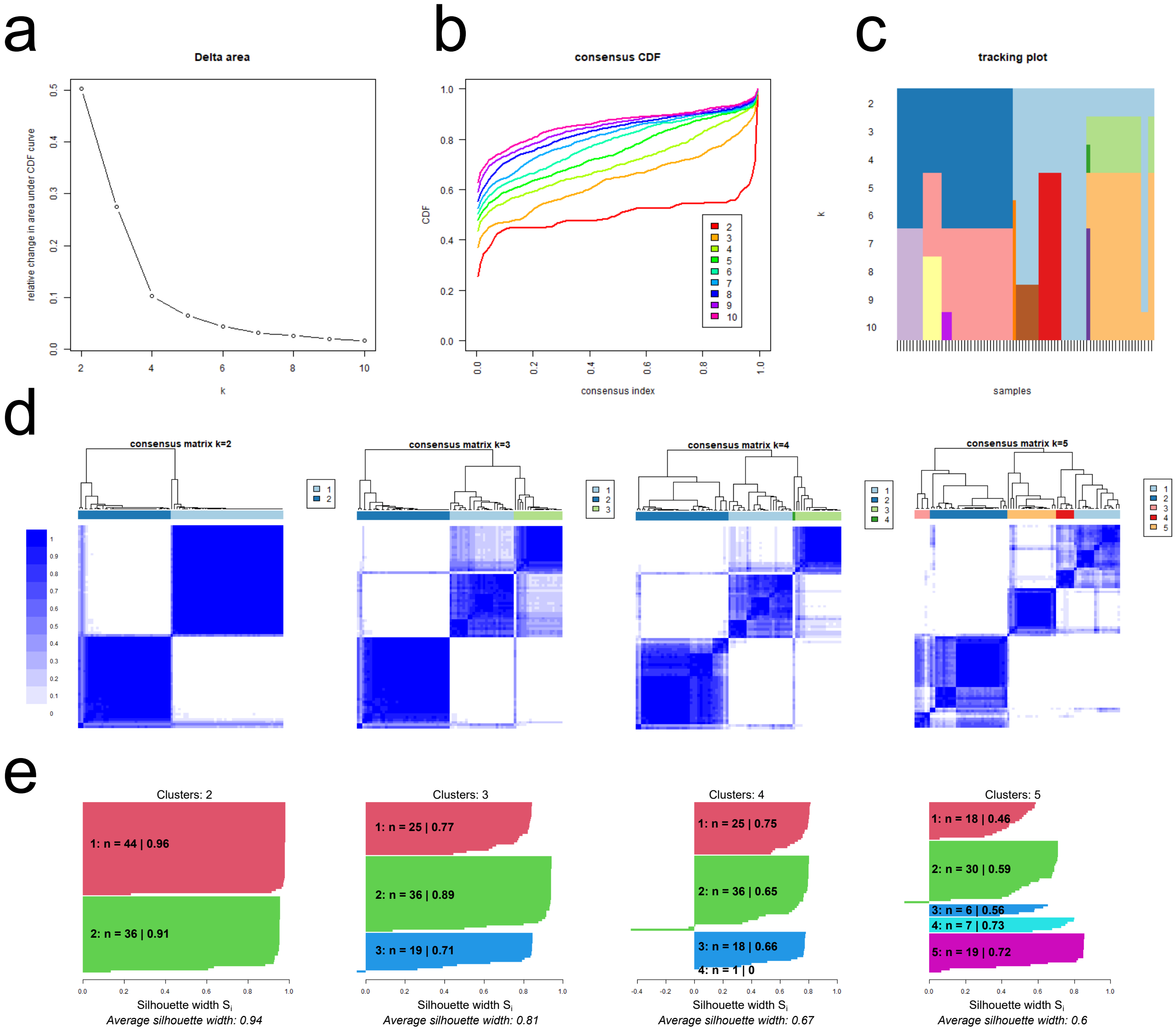

Supplementary Figure 3. Identification of leiomyosarcoma (LMS) proteomic subtypes. To investigate proteomic heterogeneity within LMS, consensus clustering (CC) was simulated for different numbers of clusters (k), and cluster stability used to determine the optimal value of k. (**a**) Delta area plot showing relative change in area under the CDF curve up to *k* (n clusters) = 10. The delta area plot showed an inflection point at k = 4, with minimal changes in the area under the curve (AUC) of the cumulative distribution function (CDF) beyond this. (**b**) Consensus CDF plot up to *k* = 10. The CDF plot showed an increase in the AUC between k = 2 and k = 3, with minor shifts in the curves beyond. (**c**) Tracking plot up to *k* = 10. The CC tracking plot indicated good cluster stability at all values of k except k = 4. At k = 4, one case was separated from the cohort, then reassigned at k = 5 to the same group as in k = 3, before being separated again at k = 7. (d) Consensus matrices up to *k* = 5. (e) Silhouette plots up to *k* = 5. Silhouette plots indicated k = 2 and k = 3 to show good clustering results with the average silhouette width (Si) for both > 0.8.

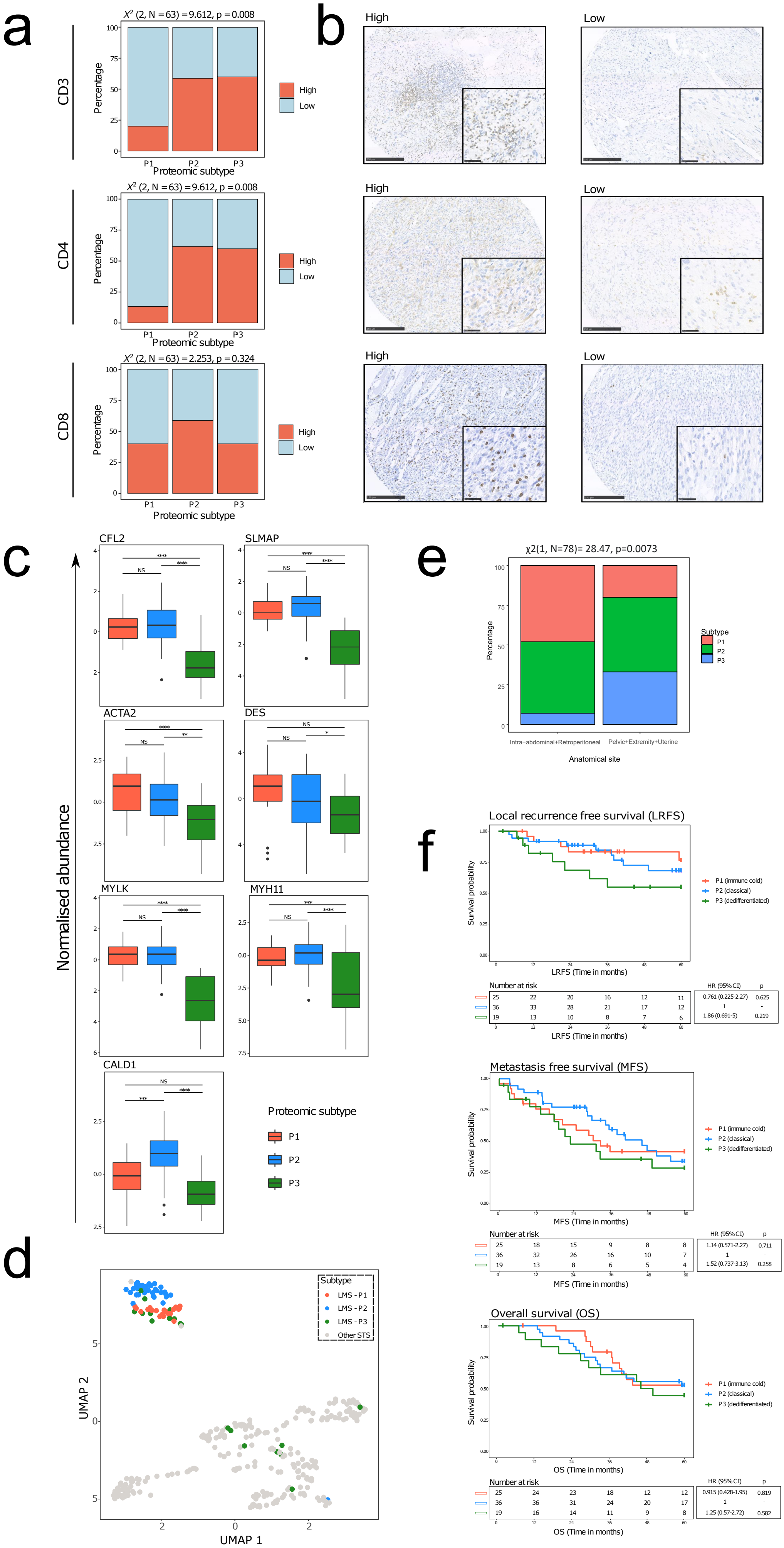

a
b
c
d
e
f
CD3
CD4
CD8
X2 (2, N =63) =9.612, p =0.008
X2 (2, N =63) =9.612, p =0.008
X2 (2, N =63) =2.253, p =0.324
Percentage
Proteomic subtype
P1
P2
P3
High
Low
CFL2
SLMAP
ACTA2
DES
MYLK
MYH11
CALD1
Normalised abundance
NS
Proteomic subtype
P1
P2
P3
UMAP 1
UMAP 2
Subtype
LMS - P1
LMS - P2
LMS - P3
Other STS
χ2(1, N=78)= 28.47, p=0.0073
Percentage
Intra−abdominal+Retroperitoneal
Pelvic+Extremity+Uterine
Anatomical site
Subtype
P1
P2
P3
Local recurrence free survival (LRFS)
Survival probability
LRFS (Time in months)
P1 (immune cold)
P2 (classical)
P3 (dedifferentiated)
Number at risk
HR (95% CI)
p
25 22 20 16 12 11
36 33 28 21 17 12
19 13 10 8 7 6
0.761 (0.225-2.27) 0.625
1 -
1.86 (0.691-5) 0.219
Metastasis free survival (MFS)
MFS (Time in months)
25 18 15 9 8 8
36 32 26 16 10 7
19 13 8 6 5 4
1.14 (0.571-2.27) 0.711
1 -
1.52 (0.737-3.13) 0.258
Overall survival (OS)
OS (Time in months)
25 24 23 18 12 12
36 36 31 24 20 17
19 16 14 11 9 8
0.915 (0.428-1.95) 0.819
1 -
1.25 (0.57-2.72) 0.582

Supplementary Figure 4. Characterisation of the biological features and clinical outcomes of leiomyosarcoma (LMS) proteomic subtypes. (a) Stacked bar plots showing the proportion of high and low CD3+/4+/8+ TILs accross each of the three proteomic subtypes of LMS (n=63). Samples were categorised as high and low based on median TIL density. Chi- square test results reported at the top of each plot. **(b)** Represenatative images of high and low CD3+, CD4+ or CD8+ TIL staining by immunohistochemistry in exemplar LMS tissue specimens (n=63). Scale bars= 250 μm (full image) and 50 μm (zoomed image). **(c)** Boxplots comparing expression of a subset of smooth muscle proteins between the three LMS subtypes (P1 n=35, P2 n=36, P3 n=19). Boxplots indicate 25th (minima), 50th (centre) and 75th percentile (maxima), with whiskers extending from 25th percentile-(1.5*IQR) to 75th percentile+(1.5*IQR), and outliers plotted as points. Significance determined by one-way analysis of variance (ANOVA) followed by Tukey's honesty significant difference (HSD) tests. NS= not significant, *p<0.05, **p<0.01, ***p<0.001, ****p<0.0001 **(d)** Uniform manifold approximation and projection (UMAP) plot showing clustering of the three LMS proteomic subtypes in relation to other soft tissue sarcomas (STS; grey) samples **(e)** Stacked bar plots showing the proportion of P1, P2 and P3 LMS subgroups in two groups of anatomical sites 1. Intra-abdominal & retroperitoneal and 2. Pelvic, extremity & uterine. Chi-square test results reported at the top of each plot. (f) Kaplan-Meier plots of overall survival (OS), metastasis free survival (MFS), and local recurrence free survival (LRFS) across the three LMS subtypes. Hazard Ratio (HR), 95% confidence intervals (CI) and p value determined by univariable Cox regression with two-sided Wald test. Source data are provided as a Source Data file.

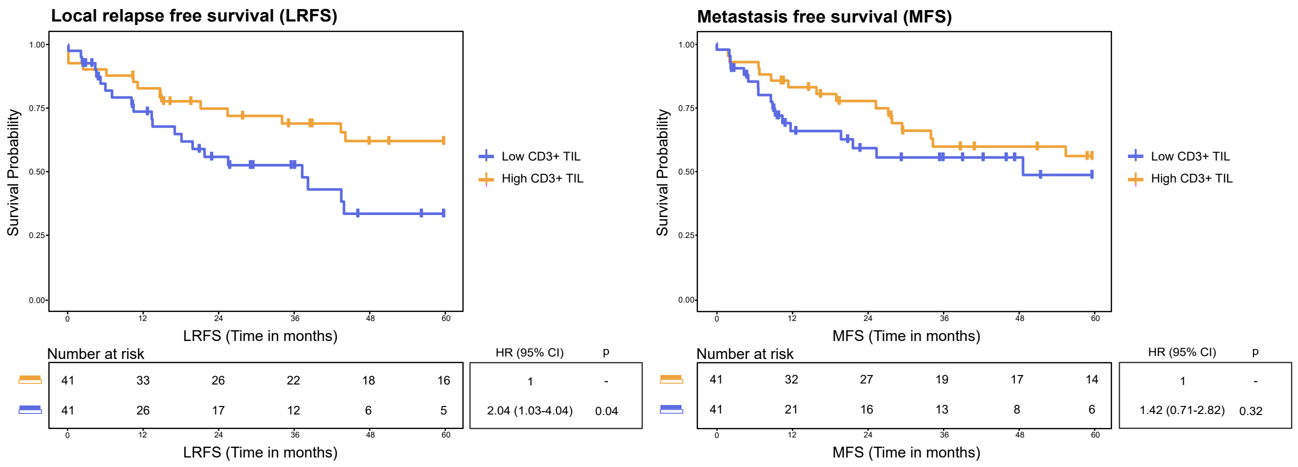

Supplementary Figure 5. Survival analysis of CD3+ TIL stratified undifferentiated pleomorphic sarcoma (UPS) and dedifferentiated liposarcoma (DDLPS) patients. Kaplan-Meier plots of local recurrence free survival (LRFS) and metastasis free survival (MFS) in high CD3+ TIL and low CD3+ TIL patients. Cases were categorised as high and low based on median TIL density. Hazard Ratio (HR), 95% confidence intervals (CI) and p value determined by univariable cox regression with two-sided Wald test.

a

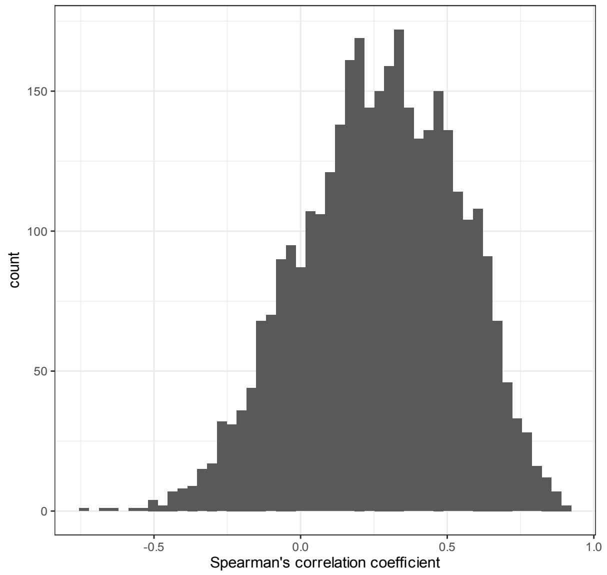

b

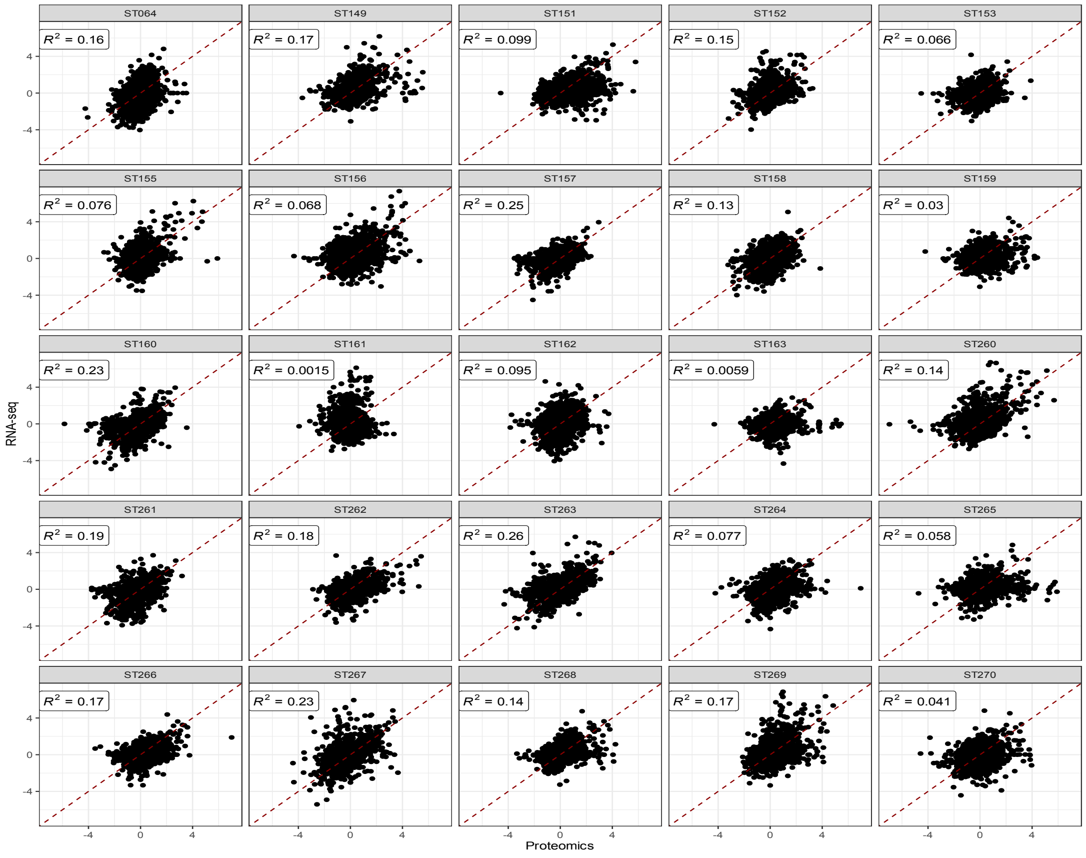

Supplemental Figure 6. Correlation analysis of RNA-seq and proteomics data in angiosarcomas (AS). (a) Histogram depicting the number of genes/proteins (y axis) with the corresponding Spearman's correlation coefficient value in the x axis. **(b)** Scatter plots of the expression values of each gene/protein in the RNA-seq (y axis) and proteomics (x axis) dataset respectively for each AS case within the cohort. R^2 indicates the coefficient of determination.

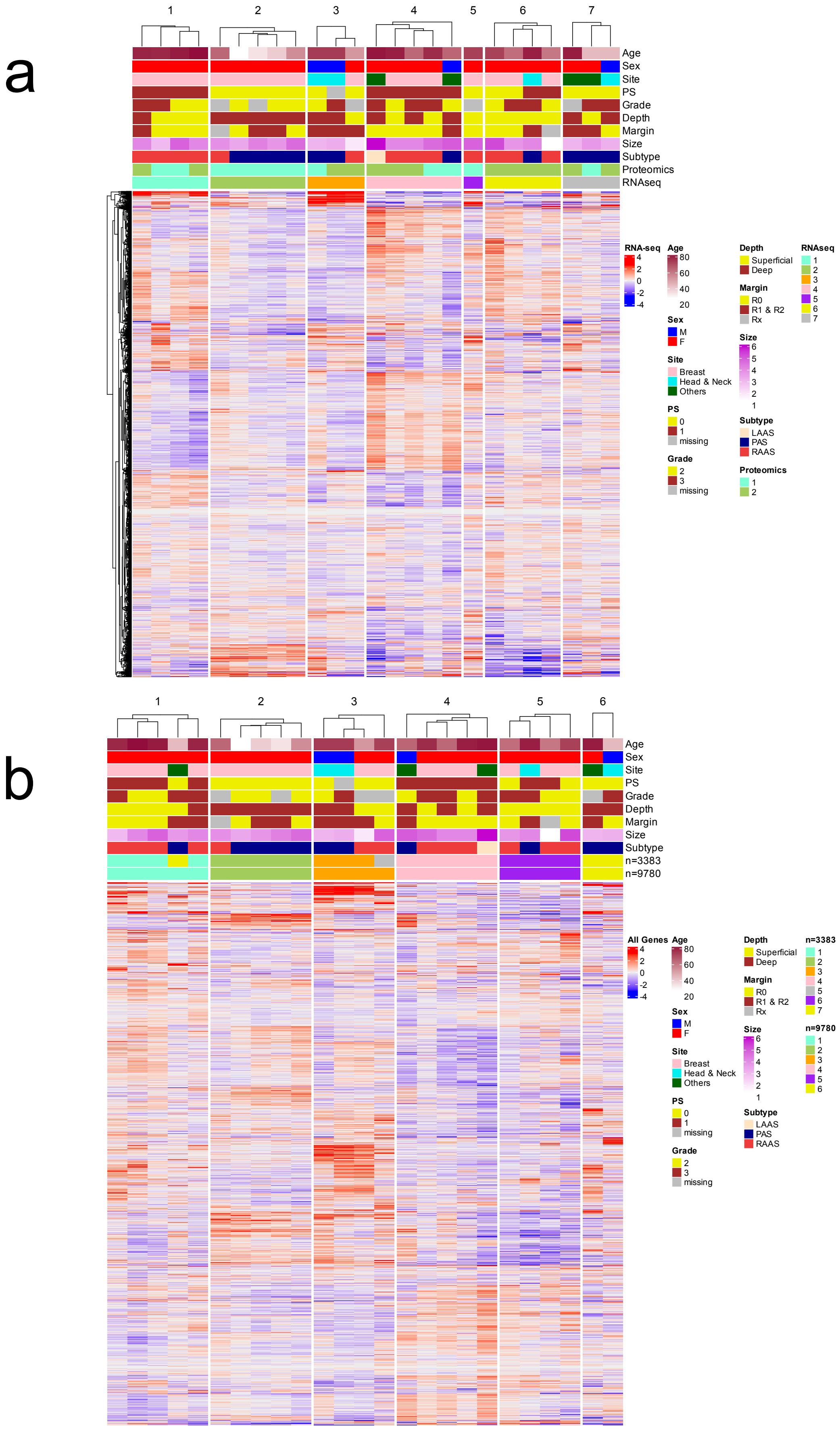

Supplementary Figure 7. Comparative analysis of RNA-seq and proteomics data in angiosarcoma (AS). (a) Annotated heatmap of RNA-seq data (3383 genes) for 25 AS cases. The samples were clustered using Monte-Carlo consensus clustering (M3C) method with K-means. From top to bottom, panels indicate age, sex, size, performance status, tumour grade, depth, margin, size and etiology/subtype. The corresponding proteomic clusters (Figure 5b) are shown. **(b)** Annotated heatmap of RNA-seq data (9780 genes) for 25 AS cases. The samples were clustered using M3C method with K-means. From top to bottom, panels indicate age, sex, size, performance status, tumour grade, depth, margin, size and etiology/subtype. The corresponding RNAseq clusters from 3383 genes (Figure S6a) are shown. Abbreviations: LAAS = lymphedema-associated angiosarcoma, PAS = primary angiosarcoma, RAAS = radiation-associated angiosarcoma, PS = performance status and Rx = margin unknown.

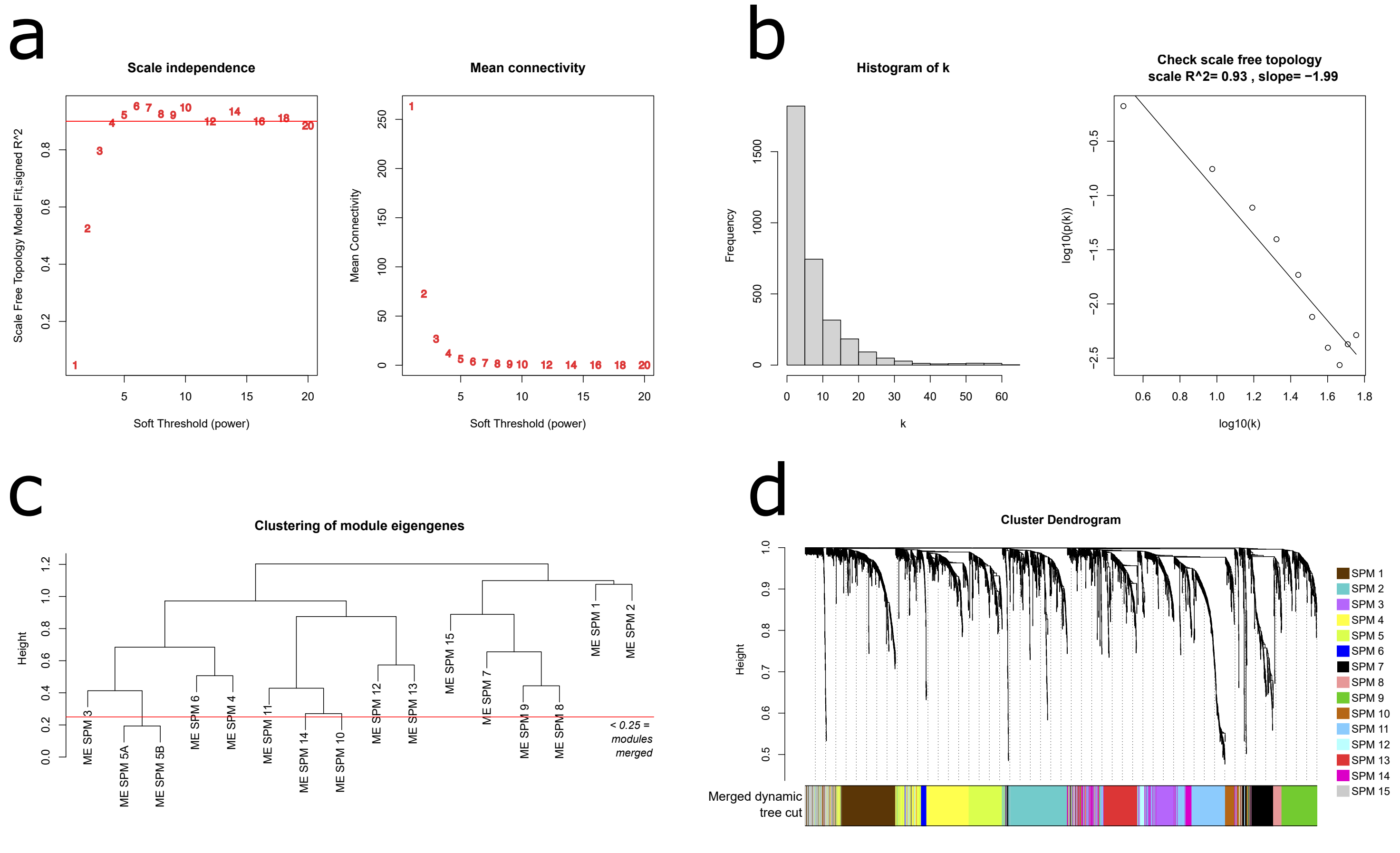

Supplementary Figure 8. Weighted gene correlation network analysis (WGCNA) for the identification of sarcoma proteome modules (SPM). (**a**) Scale free topology model fit and mean connectivity of model at Soft Threshold (power) values up to 20. Red line indicates R^2 of 0.9. (**b**) Histogram of node interactions (k) and log-log plot of k and proportion of frequency (p(k)) to assess model at a Soft Threshold (power) value of 5. (**c**) Dendrogram of SPM module eigengenes (ME) where height indicates 1-Pearson's correlation. Modules with height < 0.25 (red line) were merged. (**d**) Cluster dendrogram of all proteins where height indicates 1-Pearson's correlation. SPM identification and protein assignments by merged dynamic tree cut annotated in colour.

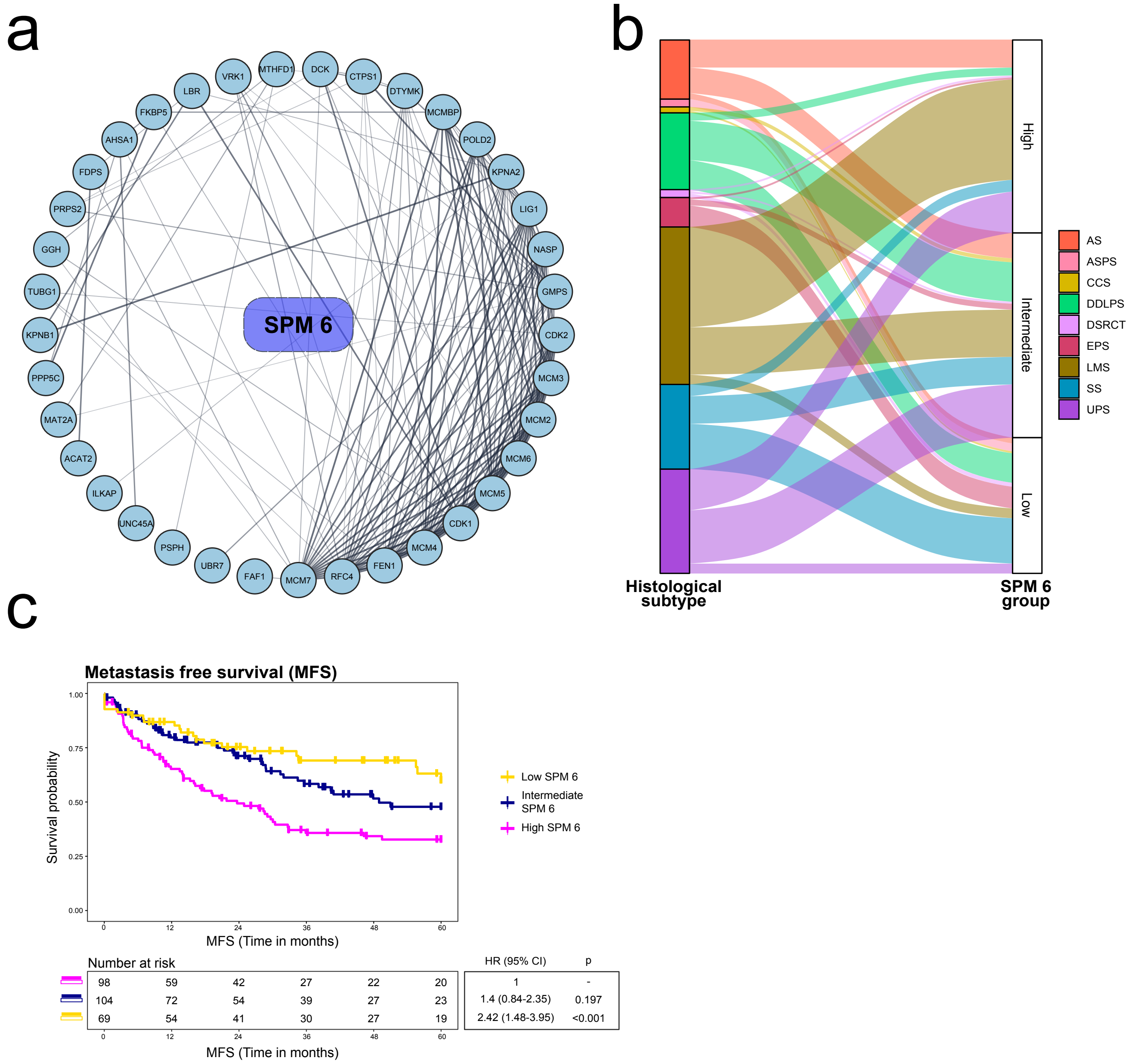

Supplementary Figure 9. Sarcoma proteome module 6 (SPM6). (a) Protein-Protein Interaction (PPI) network of SPM 6 comprising 41 nodes and 162 edges. Nodes indicate proteins and edges indicate STRING databse score between proteins, where a thicker line indicates a higher score (range = 0.402 - 0.999). **(b)** Sankey diagram illustrating the distribution of histological subtype (excluding desmoid tumour and rhabdoid tumour) across SPM 6 groups. Groups identified by tertile stratification based on median SPM 6 expression across the full cohort. **(c)** Kaplan-Meier plot of metastasis free survival (MFS) across the three SPM 6 subgroups. Hazard Ratio (HR), 95% confidence intervals (CI) and p value determined by univariable cox regression with two-sided Wald test. AS = angiosarcoma; ASPS = alveolar soft part sarcoma; CCS = clear cell sarcoma; DDLPS = dedifferentiated liposarcoma; DSRCT = desmoplastic small round cell tumour; EPS = epithelioid sarcoma; LMS = leiomyosarcoma; SS = synovial sarcoma; UPS = undifferentiated pleomorphic sarcoma.

a

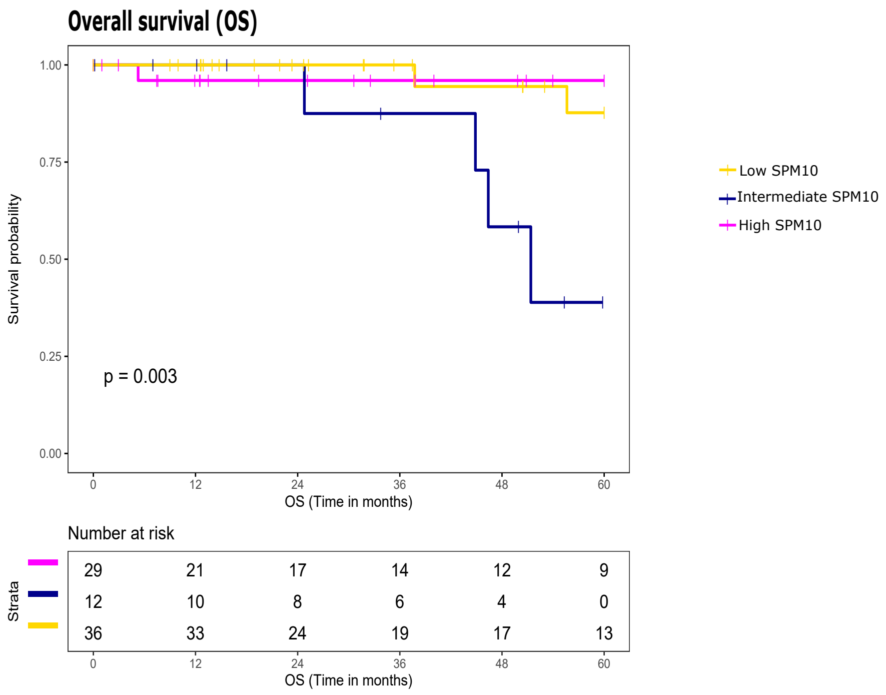

b

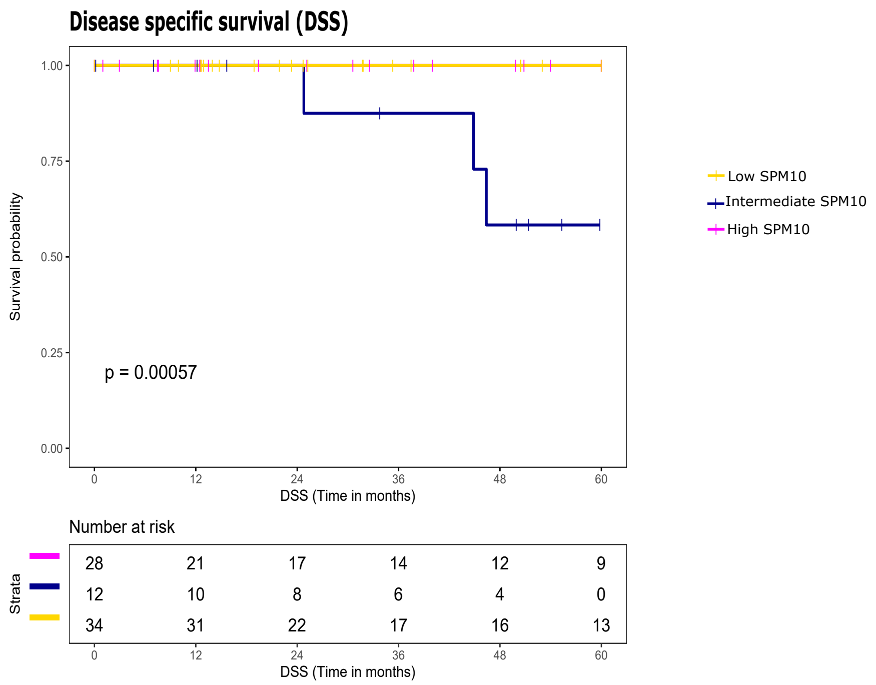

Supplementary Figure 10. Survival analysis of breast cancer patients based on application of Sarcoma proteome module 10 (SPM10) to the CPTAC proteomic dataset. Kaplan-Meier plots of (**a**) overall survival (OS) and (**b**) disease specific survival (DSS) in SPM10 high, intermediate and low breast cancer patients. p value determined by log rank test.

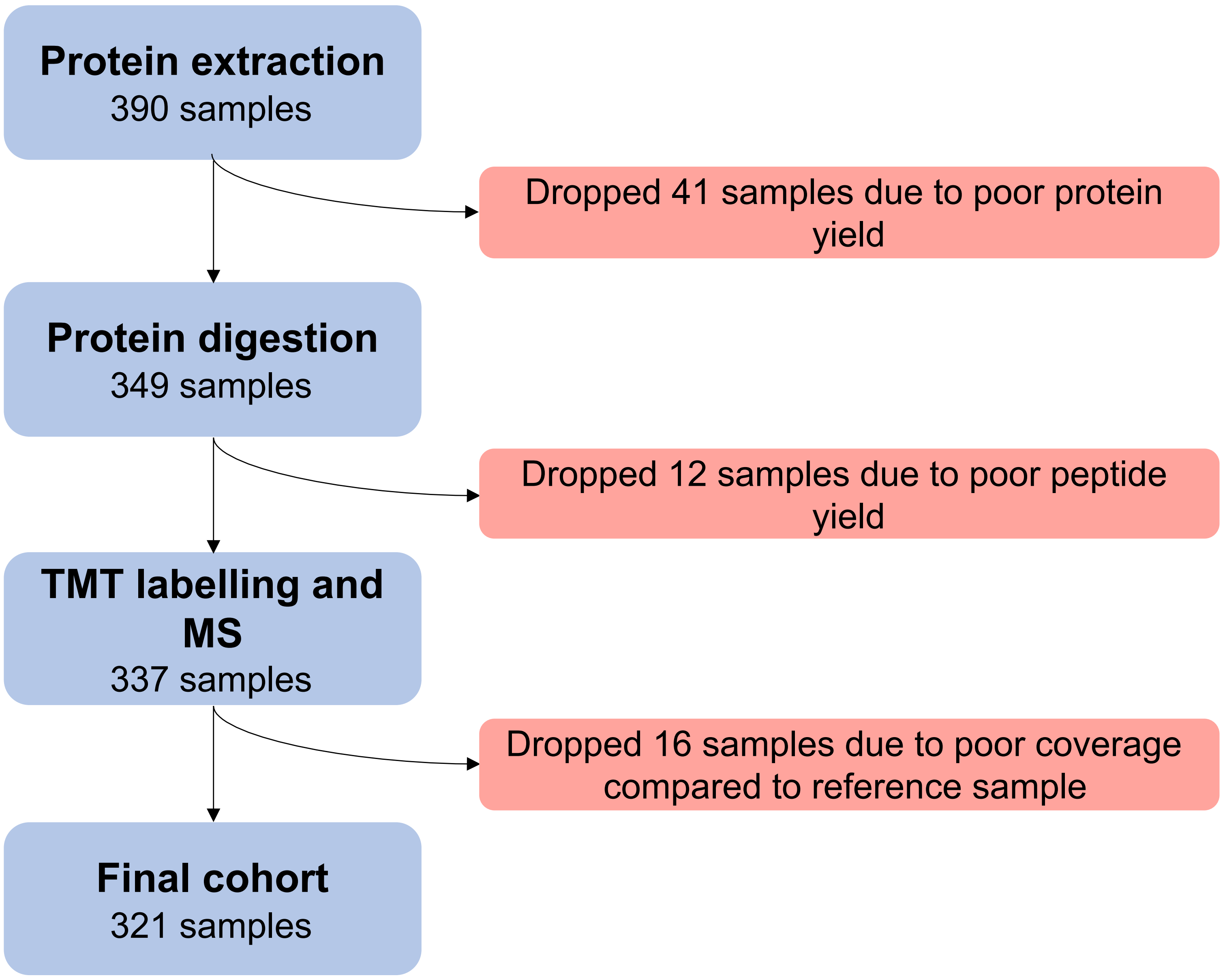

Supplementary Figure 11. Flow diagram indicating the drop-out rate of samples following quality control. Of a total of 390 samples that were retrieved from our archive, 41 samples were dropped due to poor protein extraction yields from lysis, 12 samples dropped due to poor peptide yields following protein digestion and 16 samples dropped dueto poor proteome coverage following MS data acquisition, resulting in a final cohort of 321cases.

Table S1. Associations between leiomyosarcoma (LMS) proteomic subtypes and clinicopathological features.

		LMS subtype			Test results			
	**Variable**	**P1 (immune cold) n = 25**	**P2 (classical) n = 36**	**P3 (dedifferentiated) n = 19**	**Test performed**	**χ-squared**	**Degrees of freedom**	**p value**
**Age at excision (years)**	**median**	61.5	66.8	65.4	Kruskal Wallis	0.373	2	0.83
	**min**	31.4	30.5	29.3				
	**max**	83.6	86.9	83.5				
**Tumour size (mm)**	**median**	100	80	110	Kruskal Wallis	3.131	2	0.209
	**min**	50	25	5				
	**max**	400	290	250				
**Sex [n (%)]**	**F**	18 (72.0)	26 (72.2)	12 (63.2)	Chi-square	0.556	2	0.757
	**M**	7 (28.0)	10 (27.8)	7 (36.8)				
**Grade [n (%)]**	**2**	15 (60.0)	21 (58.3)	11 (57.9)	Chi-square	0.024	2	0.988
	**3**	10 (40.0)	15 (41.7)	8 (42.1)				
**Performance status [n (%)]**	**0**	15 (60.0)	17 (47.2)	8 (42.1)	Chi-square	13.304	8	0.102
	**1**	2 (8.0)	12 (33.3)	2 (10.5)				
	**2**	2 (8.0)	3 (8.3)	2 (10.5)				
	**3**	-	1 (2.7)	-				
	**unknown**	6 (24.0)	3 (8.3)	7 (36.8)				
**Pre-op treatment [n (%)]**	**Radio**	-	-	1 (5.3)	Chi-square	3.251	2	0.197
	**None**	25 (100.0)	36 (100.0)	18 (94.7)				
**Anatomical site [n (%)]**	**Extremity**	7 (28.0)	14 (38.9)	10 (52.6)	Chi-square	12.032	10	0.283
	**Intra-abdominal**	5 (20.0)	4 (11.1)	1 (5.3)				
	**Pelvic**	1 (4.0)	5 (13.9)	3 (15.8)				
	**Retroperitoneal**	9 (36.0)	9 (25.0)	1 (5.3)				
	**Trunk**	1 (4.0)	-	1 (5.3)				
	**Uterine**	2 (8.0)	4 (11.1)	3 (15.8)				
**Status at excision [n (%)]**	**Local**	24 (96.0)	36 (100.0)	18 (94.7)	Chi-square	1.749	2	0.417
	**Metastatic**	1 (4.0)	-	1 (5.3)				
**Tumour depth [n (%)]**	**Deep**	22 (88.0)	28 (77.8)	16 (84.2)	Chi-square	1.118	2	0.572
	**Superficial**	3 (12.0)	8 (22.2)	3 (15.8)				
**Tumour margins [n (%)]**	**R0**	13 (52.0)	20 (58.8)	9 (47.4)	Chi-square	5.342	6	0.501
	**R1**	11 (44.0)	14 (41.2)	10 (52.6)				
	**R2**	1 (4.0)	-	-				

Table S2. Univariable and multivariable cox regression assessing local recurrence free survival (LRFS) in patients categorised by leiomyosarcoma (LMS) proteomic subtype. HR = hazard ratio; CI = confidence interval.

			**Multivariable analysis (LRFS)**	
	**Variable**	**n**	**HR (95% CI)**	**p value**
	**Age (years)**	**-**	**0.956 (0.918-0.996)**	**0.031**
**Log[tumour size] (mm)**	***4-5 (reference)***	50	-	-
	**<4**	12	1.18 (0.158-8.73)	0.875
	**>5**	16	0.538 (0.14-2.07)	0.368
**Sex**	***F (reference)***	54	-	-
	**M**	24	**5.54 (1.54-20)**	**0.009**
**Grade**	***2 (reference)***	45	-	-
	**3**	33	2.89 (0.926-9)	0.068
**Performance status**	***0 (reference)***	38	-	-
	**1**	16	**7.82 (1.74-35)**	**0.007**
	**2-3**	8	5.52 (0.334-91.1)	0.233
	**unknown**	16	1.59 (0.361-6.96)	0.542
**Anatomical site**	***I-A/RP/Pelvic (reference)***	37	-	-
	**Extremity/Trunk wall**	33	**0.089 (0.018-0.452)**	**0.004**
	**Uterine**	8	0.148 (0.01-2.29)	0.172
**Tumour margin**	***R0 (reference)***	42	-	-
	**R1&R2**	36	1.8 (0.628-5.14)	0.274
**Tumour depth**	***Deep (reference)***	64	-	-
	**Superficial**	14	0.514 (0.036-7.41)	0.625
**LMS subtype**	***P2 (reference)***	34	-	-
	P1	25	1.19 (0.308-4.61)	0.8
	**P3**	19	**8.04 (1.7-38)**	**0.009**

Table S3. Multivariable cox regression assessing overall survival (OS) of undifferentiated pleomorphic sarcoma (UPS) & dedifferentiated liposarcoma (DDLPS) patients categorised as CD3+ tumour infiltrating lymphocyte (TIL) high and low. HR = hazard ratio; CI = confidence interval; RP = retroperitoneal; IA = intra-abdominal.

			Multivariable analysis (OS)	
**Variable**		**n**	**HR (95% CI)**	**p value**
**Age (years)**		-	1.04 (1.01-1.09)	0.0266
**Log[tumour size] (mm)**	***4-5 (reference)***	36	-	-
	**<4**	14	0.325 (0.103-1.02)	0.054
	**>5**	32	1.75 (0.718-4.26)	0.219
**Sex**	***M (reference)***	46	-	-
	**F**	36	1.32 (0.668-2.62)	0.423
**Grade**	***3 (reference)***	63	-	-
	**2**	18	0.618 (0.238-1.6)	0.323
	**unknown**	1	9.77 (0.874-109)	0.064
**Performance status**	***0 (reference)***	39	-	-
	**1**	16	2.16 (0.914-5.09)	0.079
	**2-3**	7	1.98 (0.58-6.76)	0.275
	**unknown**	16	1.92 (0.62-5.95)	0.258
**Anatomical site**	***Other (reference)***	31	-	-
	**RP/IA**	9	1.59 (0.316-8.01)	0.574
**Tumour margin**	***R1 & R2 (reference)***	40	-	-
	**R0**	35	0.911 (0.421-1.97)	0.813
	**unknown**	3	0.774 (0.079-7.55)	0.825
**Tumour depth**	***Deep (reference)***	64	-	-
	**Superficial**	14	0.807 (0.231-2.82)	0.738
**Histological subtype**	***UPS (reference)***	35	-	-
	**DDLPS**	25	0.559 (0.119-2.62)	0.461
**CD3+ TIL**	***High (reference)***	35	-	-
	**Low**	25	2.07 (1.01-4.23)	**0.048**

Table S4 Multivariable cox regression assessing metastasis free survival (MFS) in patients categorised by sarcoma proteome module (SPM) 10 median expression levels. HR = hazard ratio; CI = confidence interval.

			**Multivariable analysis (MFS)**	
	**Variable**	**n**	**HR (95% CI)**	**p value**
	**Age (years)**	-	0.996 (0.981-1.01)	0.581
**Histological subtype**	***Leiomyosarcoma (reference)***	78	-	-
	**Angiosarcoma**	30	2.72 (1.42-5.21)	**0.003**
	**Dedifferentiated liposarcoma**	39	0.359 (0.16-0.805)	**0.013**
	**Epithelioid sarcoma**	14	3.51 (1.34-9.19)	**0.011**
	**Synovial sarcoma**	43	0.697 (0.329-1.48)	0.347
	**Undifferentiated pleomorphic sarcoma**	53	0.889 (0.502-1.57)	0.685
	**Other**	11	1.53 (0.514-4.55)	0.445
**Log[tumour size] (mm)**	***4-5(reference)***	138		
	**<4**	65	0.43 (0.247-0.749)	**0.003**
	**>5**	65	1.02 (0.616-1.68)	0.974
**Sex**	***F (reference)***	163	-	-
	**M**	105	1.33 (0.867-2.03)	0.192
**Grade**	***2 (reference)***	112	-	-
	3	138	2.26 (1.47-3.47)	**<0.001**
	**unknown**	18	0.859 (0.317-2.33)	0.764
**Performance status**	***0 (reference)***	129	-	-
	**1**	77	1.65 (1.02-2.66)	**0.041**
	**2-3**	20	1.27 (0.563-2.85)	0.567
	**unknown**	42	1.39 (0.808-2.37)	0.236
**Tumour margin**	***R1&R2 (reference)***	133	-	-
	**R0**	121	1.12 (0.76-1.66)	0.557
	**unknown**	14	1.17 (0.406-3.38)	0.77
**Tumour depth**	***Deep (reference)***	216	-	-
	**Superficial**	52	0.471 (0.262-0.848)	**0.012**
**SPM 10 expression**	***Low***	105	-	-
	**Intermediate**	91	0.79 (0.481-1.3)	0.352
	**High**	72	0.46 (0.249-0.847)	**0.013**

Supplemental methods

Comparative analysis of angiosarcoma cohort

Tumour total RNA was extracted using the High Pure miRNA kit (Roche, Penzberg, Germany) following vendor's standard protocol. mRNA concentrations were measured using Qubit fluorometric quantitation (Thermo Fisher Scientific, Waltham, MA, USA). RNA Integrity Number was measured using 2100 Bioanalyzer system (Agilent, CA, USA). RNA samples were stored at -80°C until use.

RNA-seq and analysis
At least 500 ng was used for exon-capture based RNA-seq library preparation using the SureSelect XT RNA Direct kit and the SureSelect Exome V6+UTR Capture kit (Agilent, Santa Clara, CA). Libraries were sequenced on the NovaSeq platform (Illumina, San Diego, CA). Salmon was used for quantifying the expression of transcripts[1] based on gencode version 22 GTF transcript annotation[2]. The filterByExpr function in edgeR[3] was used to determine expressed genes

RNA-seq data pre-processing
The raw RNA-seq fastq files were pre-processed and normalised following the steps below:

1. From fastq files, adapter sequences were removed with trim-galore (v0.6.6) before alignment to the genome.
2. Trimmed fastq reads were aligned to the genome and raw counts were generated with Salmon (v1.3.0) using gencode v22 annotation.
3. The variance stabilizing transformation function from DESeq2 (v1.36) was used to generate log2 transformed counts.
4. The normalised RNA-seq counts were then median centred across the 25 samples.

Proteomics data pre-processing
The proteomics data was processed using the following steps:
1. Proteins identified in < 75% of the samples (n=25) were removed, and those remaining imputed using the k-nearest neighbour (KNN) algorithm.
2. Each sample was divided by the corresponding reference sample.
3. Data was then Log2 transformed.
4. Z-score standardised per sample (column standardisation).
5. And median centred per protein (row median centring).

Matching proteins (proteomics dataset) with genes (RNA-seq dataset)
In the proteomics dataset, the identifiers for the genes/proteins were in the form of gene symbols. For the RNA-seq dataset, the identifiers for the genes were in the form of Ensembl gene ID, with corresponding gene symbols provided. The following steps were followed to match the proteins with genes:
1. Gene symbols from the proteomics dataset were converted to Ensembl gene ID via gProfiler[4]. 27 genes returned 2 different Ensembl gene IDs.
2. For these 27 genes, the following was removed:
    a. Long non-coding RNA, novel transcript/protein, and readthrough genes
    b. Genes that returned a different name than the original input
3. After step 2, both TBCE and MATR3 had two Ensembl IDs associated. Genecards.org was used to identify the correct ID.
4. Of the remaining 3393 Ensembl IDs, 10 were not found in the RNA-seq dataset, these genes were: H1-0, IGHV3-43D, RNASE3, GSTT1, HLA-DRB3, H2BC21, FAHD1, PI4K2A, BOLA2, and AKAP2.

In summary, 3383 genes/proteins were present in both Proteomics and RNA-seq datasets.

RNA-seq global gene list
In total, the expression of 59987 genes were captured in the RNA-seq dataset. We selected 9780 genes from these 59987 genes to form the global gene list following two criteria:

1. We selected genes with variance > 0.5 (n = 7558) across the 25 samples.
2. We selected genes with variance ≤ 0.5 but were present in both Proteomics and RNA-seq dataset (n = 2222).

Statistical analysis
Statistical analysis was performed using the R software (version 4.1.2). P-values were considered significant if lower than 0.05 and multiple testing correction was performed using the Benjamini & Hochberg (FDR) method[5]. Spearman Rank correlation was used to calculate the correlations between gene expressions and protein expressions. Monte-Carlo reference-based consensus clustering (M3C)[6] with K-Means as the core clustering algorithm (0.8 sampling rate per iteration for 25 iterations) was applied to identify clusterships.

Univariable cox regression models were fit to identify gene/protein expressions that were significantly associated with clinical outcomes (OS, MFS and LRFS). Multivariable cox regression models were adjusted for clinicopathological variables including grade, tumour depth and tumour size. Missing data were handled by either excluding patients with missing data from the analyses or incorporating the missing value as a separate level of the variable. In this angiosarcoma cohort, 5 patients had a missing grade. Changes in log-likelihood ratio (chi-square) were used to evaluate the additional prognostic ability provided by specific variables that were added to the multivariable cox regression models.

References

1. Patro, R., Duggal, G., Love, M.I., Irizarry, R.A. & Kingsford, C. Salmon provides fast and bias-aware quantification of transcript expression. *Nat Methods* **14**, 417-419 (2017).

2. McCarthy, D.J., Chen, Y. & Smyth, G.K. Differential expression analysis of multifactor RNA-Seq experiments with respect to biological variation. *Nucleic Acids Res* **40**, 4288-4297 (2012).

3. Love, M.I., Huber, W. & Anders, S. Moderated estimation of fold change and dispersion for RNA-seq data with DESeq2. *Genome Biol* **15**, 550 (2014).

4. Raudvere, U.*, et al.* g:Profiler: a web server for functional enrichment analysis and conversions of gene lists (2019 update). *Nucleic Acids Res* **47**, W191-W198 (2019).

5. Benjamini, Y. & Hochberg, Y. Controlling the False Discovery Rate: A Practical and Powerful Approach to Multiple Testing. *Journal of the Royal Statistical Society: Series B (Methodological)* **57**, 289-300 (1995).

6. John, C.R.*, et al.* M3C: Monte Carlo reference-based consensus clustering. *Sci Rep* **10**, 1816 (2020).
