# Supplementary material for: The proteomic landscape of soft tissue sarcomas
Source: Nat Commun. 2023 Jun 29;14:3834. doi: 10.1038/s41467-023-39486-2 (PMC10310735; doi:10.1038/s41467-023-39486-2)
Supplement: Supplementary file 2 — Description of Additional Supplementary Files [file 41467_2023_39486_MOESM2_ESM.pdf]

Description of Additional Supplementary Files

Supplementary Data 1. Clinicopathological features of the study cohort.

Supplementary Data 2. Normalised proteomic profiling data.

Supplementary Data 3 a-f. Significantly upregulated unique proteins in (a)angiosarcoma (AS), (b) dedifferentiated liposarcoma (DDLPS), (c) desmoid tumour (DES), (d) leiomyosarcoma (LMS), (e) synovial sarcoma (SS), and (f) undifferentiated pleomorphic sarcoma (UPS). Proteins were identified by 2-class unpaired significance analysis of microarray (SAM) analysis (FDR < 1%, fold change ≥1.5).

Supplementary Data 4 a-d. Overrepresentation analysis results for proteins uniquely upregulated in (a) angiosarcoma (AS), (b) desmoid tumour (DES), (c) leiomyosarcoma (LMS), and (d) undifferentiated pleomorphic sarcoma (UPS). p values were adjusted for multiple testing using the Benjamini & Hochberg (FDR) method. Significance determined at p.adjust < 0.05.

Supplementary Data 5a-c. Significantly upregulated unique proteins in (a) synovial sarcoma (SS) compared to other subtypes, and in (b) untreated SS (SSu) and (c) treated SS (SSt) compared to other subtypes. Proteins were identified by 2-class unpaired significance analysis of microarray (SAM) analysis (FDR < 1%, fold change ≥1.5).

Supplementary Data 6a-b. Associations between leiomyosarcoma (LMS) proteomic subtypes and ssGSEA hallmark gene set results. (a) One-way analysis of variance (ANOVA) results with p values adjusted to false discovery rate (FDR) using the Benjamini-Hochberg procedure to account for multiple comparisons. (b) Post-hoc Tukey's honestly significant differences (HSD) results.

Supplementary Data 7. List of 153 proteins used in leiomyosarcoma proteomic subtype classification.

Supplementary Data 8. Normalised NanoString PlexSet Immune profiling data.

Supplementary Data 9. Gene set enrichment analysis results for undifferentiated pleomorphic sarcoma (UPS) & dedifferentiated liposarcoma (DDLPS) cases. Proteins ranked based on CD3+ tumour infiltrating lymphocyte (TIL) high-low fold change. p values were calculated by permutation and adjusted using the Benjamini-Hochberg procedure to account for multiple comparisons. Significance determined at p.adjust < 0.05. Top and bottom 15 sets based on normalised enrichment score are shown.

Supplementary Data 10. Sarcoma proteome module (SPM) membership.

Supplementary Data 11. List of 53 proteins used in SPM10 subgroup classification.
