# Supplementary material for: The proteomic landscape of soft tissue sarcomas
Source: Nat Commun. 2023 Jun 29;14:3834. doi: 10.1038/s41467-023-39486-2 (PMC10310735; doi:10.1038/s41467-023-39486-2)
Supplement: Supplementary file 14 — Reporting Summary [file 41467_2023_39486_MOESM14_ESM.pdf]

nature portfolio

Corresponding author(s): Paul Huang

Last updated by author(s): Jun 12, 2023

Reporting Summary

Nature Portfolio wishes to improve the reproducibility of the work that we publish. This form provides structure for consistency and transparency in reporting. For further information on Nature Portfolio policies, see our Editorial Policies and the Editorial Policy Checklist.

Statistics

For all statistical analyses, confirm that the following items are present in the figure legend, table legend, main text, or Methods section.

n/a	Confirmed	
☐	☒	The exact sample size (*n*) for each experimental group/condition, given as a discrete number and unit of measurement
☒	☐	A statement on whether measurements were taken from distinct samples or whether the same sample was measured repeatedly
☐	☒	The statistical test(s) used AND whether they are one- or two-sided<br>*Only common tests should be described solely by name; describe more complex techniques in the Methods section.*
☐	☒	A description of all covariates tested
☐	☒	A description of any assumptions or corrections, such as tests of normality and adjustment for multiple comparisons
☐	☒	A full description of the statistical parameters including central tendency (e.g. means) or other basic estimates (e.g. regression coefficient) AND variation (e.g. standard deviation) or associated estimates of uncertainty (e.g. confidence intervals)
☐	☒	For null hypothesis testing, the test statistic (e.g. *F*, *t*, *r*) with confidence intervals, effect sizes, degrees of freedom and *P* value noted<br>*Give P values as exact values whenever suitable.*
☒	☐	For Bayesian analysis, information on the choice of priors and Markov chain Monte Carlo settings
☒	☐	For hierarchical and complex designs, identification of the appropriate level for tests and full reporting of outcomes
☐	☒	Estimates of effect sizes (e.g. Cohen's *d*, Pearson's *r*), indicating how they were calculated

Our web collection on statistics for biologists contains articles on many of the points above.

Software and code

Policy information about availability of computer code

Data collection	Mass spectrometry data was collected on an Orbitrap Fusion Lumos Mass Spectrometer.<br>TCGA SARC RPPA data was downloaded from The Cancer Proteome Atlas portal (https://tcpaportal.org/tcpa/) and clinical data downloaded from the TCGA Pan-cancer Clinical Data Resource (TCGA-CDR) within the NCI Genomic Data Commons (https://gdc.cancer.gov/about-data/publications/PanCan-Clinical-2018).<br>NanoString gene expression data was collected on the nCounter PlexSet-96 platform using a custom gene panel.
Data analysis	Mass spectrometry data was searched using Proteome Discoverer (v2.2 or v2.3).<br>Protein-protein interaction network analysis was performed using Cytoscape (v3.9.1) and the STRING database (v11).<br>Single sample gene set enrichment analysis was performed with the ssGSEA (v10.0.11) module on the GenePattern public server.<br>The remaining downstream analyses were performed in R (v3.5.1 or later), using packages: tidyverse, EnvStats, matrixStats, ggplot2, ggpubr, circlize, scales, RColorBrewer, msigdbr, enrichplot, DOSE, org.Hs.eg.db, impute, umap, ConsensusClusterPlus, cluster, samr, ClusterProfiler, WGCNA, survival, survminer, ComplexHeatmap, NanoStringNorm.<br><br>No custom algorithms were written as part of this analysis and no custom software was developed.

For manuscripts utilizing custom algorithms or software that are central to the research but not yet described in published literature, software must be made available to editors and reviewers. We strongly encourage code deposition in a community repository (e.g. GitHub). See the Nature Portfolio guidelines for submitting code & software for further information.

Data

Policy information about availability of data

All manuscripts must include a data availability statement. This statement should provide the following information, where applicable:

- Accession codes, unique identifiers, or web links for publicly available datasets
- A description of any restrictions on data availability
- For clinical datasets or third party data, please ensure that the statement adheres to our policy

The raw proteomic data generated in this study have been deposited in the ProteomeXchange Consortium via the PRIDE partner repository97,98 with the dataset identifier PXD036226 [https://www.ebi.ac.uk/pride/archive/projects/PXD036226]. The raw transcriptomic data are deposited at the European Genome-phenome Archive (EGA)99, which is hosted by the EBI and the CRG, under accession number EGAD00001010839 [https://ega-archive.org/datasets/EGAD00001010839]. To protect patient privacy, as required by law, access to the raw transcriptomic data deposited in the EGA is controlled by the Data Access Committee (DAC) of the Institute of Cancer Research. All researchers can obtain access by submitting a project proposal to the DAC by contacting the corresponding author (P.H.H.). Requests will be handled within ~2 weeks. The DAC will also determine the length of permitted access. The clinical data is available under restricted access due to data privacy legislation, access can be obtained by contacting the corresponding author (P.H.H) and will require the researcher to sign a data access agreement with the Institute of Cancer Research after approval by the DAC. The DAC will determine the length of permitted access with an expected response timeframe of ~2 weeks for access requests. The normalised proteomic dataset and normalised NanoString dataset are provided in the Supplementary Information. The TCGA SARC RPPA data is available from The Cancer Proteome Atlas portal (https://tcpaportal.org/tcpa/) and clinical data is available from the TCGA Pan-cancer Clinical Data Resource (TCGA-CDR) within the NCI Genomic Data Commons (https://gdc.cancer.gov/about-data/publications/PanCan-Clinical-2018). The raw mass spectra was searched against UniProt human protein entries (v2018_07 or later) for protein identification and quantification (https://www.uniprot.org/proteomes/UP000005640). Source data are provided with this paper.

Human research participants

Policy information about studies involving human research participants and Sex and Gender in Research.

Reporting on sex and gender	Data on patient sex (assigned) was collected as part of this study and was used as a covariate in analyses throughout this manuscript. Out of 321 patients, 201 were assigned female at birth (63%) and 119 male at birth (37%). Information on gender was not collected.
Population characteristics	Patients with a histopathologically confirmed diagnosis of soft tissue sarcoma (STS) or desmoid tumour were selected for inclusion based on the availability of sufficient primary tumour tissue in institutional archives. STS diagnoses of angiosarcoma, alveolar soft part sarcoma, clear cell sarcoma, dedifferentiated liposarcoma, desmoplastic small round cell tumour, epithelioid sarcoma, synovial sarcoma, leiomyosarcoma, rhabdoid tumour, and undifferentiated pleomorphic sarcoma were included. Patient characteristics were typical of a multi-subtype primary STS cohort (median age = 58, 83% were treatment naive). Full population characteristics can be found in Supplemental Table 1.
Recruitment	This is a retrospective tissue-based study and therefore prospective recruitment is not applicable.
Ethics oversight	This study was approved as part of the Royal Marsden Hospital (RMH) PROgnoStic and PrEdiCTive ImmUnoprofiling of Sarcomas (PROSPECTUS) study (NHS Research Ethics Committee Reference 16/EE/0213), National Taiwan University Hospital (Research Ethics Committee Reference 201912226RINB), and Newcastle University as part of Children's Cancer and Leukaemia Group (CCLG) Biological Study 2012 BS 05 (Research Ethics Committee Reference 8/EM/0134). Written informed consent was obtained from participants.

Note that full information on the approval of the study protocol must also be provided in the manuscript.

Field-specific reporting

Please select the one below that is the best fit for your research. If you are not sure, read the appropriate sections before making your selection.

☒ Life sciences ☐ Behavioural & social sciences ☐ Ecological, evolutionary & environmental sciences

For a reference copy of the document with all sections, see nature.com/documents/nr-reporting-summary-flat.pdf

Life sciences study design

All studies must disclose on these points even when the disclosure is negative.

Sample size	321 samples were included based on sample availability. Sample size is sufficient due to the rarity of the disease. Furthermore, this is the largest STS cohort to undergo proteomic profiling to date.
Data exclusions	Rhabdoid tumour (RT) and desmoid tumour (DES) patients were excluded from survival analyses. The reason for this exclusions are that the survival endpoints of overall survival (OS), progression free survival (PFS) and local relapse free survival (LRFS) are not relevant to these two disease types as outlined below.<br>RT are paediatric tumours which when compared to adult STS show distinct clinical patterns and undergo different management.

DES are locally aggressive and show no metastatic potential and therefore OS, PFS and LRFS are not clinically meaningful measures of disease outcome and not comparable to the rest of the STS cohort.

Replication	Proteomic and transcriptomic measurements were taken from individual samples within the cohort, no technical replicates were performed.
Randomization	Randomization is not relevant as this is a retrospective tissue-based study.
Blinding	Blinding is not relevant, as samples were not allocated to experimental groups.

Reporting for specific materials, systems and methods

We require information from authors about some types of materials, experimental systems and methods used in many studies. Here, indicate whether each material, system or method listed is relevant to your study. If you are not sure if a list item applies to your research, read the appropriate section before selecting a response.

Materials & experimental systems

n/a	Involved in the study
☐	☒ Antibodies
☒	☐ Eukaryotic cell lines
☒	☐ Palaeontology and archaeology
☒	☐ Animals and other organisms
☒	☐ Clinical data
☒	☐ Dual use research of concern

Methods

n/a	Involved in the study
☒	☐ ChIP-seq
☒	☐ Flow cytometry
☒	☐ MRI-based neuroimaging

Antibodies

Antibodies used	CD3 Agilent DAKO A0452<br>CD4 Agilent DAKO 4B12<br>CD8 Agilent DAKO C8/144B<br>Secondary antibody: DAKO FlexEnvision (Mouse) Kit, followed by application of DAB and haematoxylin counterstaining.
Validation	These antibodies are well established CE-marked IVD antibodies which are routinely used. Details for validation are provided in the following manufacturer links.<br>https://www.agilent.com/en/product/immunohistochemistry/antibodies-controls/primary-antibodies/cd3-(concentrate)-76133<br>https://www.agilent.com/en/product/immunohistochemistry/antibodies-controls/primary-antibodies/cd4-(concentrate)-76673<br>https://www.agilent.com/en/product/immunohistochemistry/antibodies-controls/primary-antibodies/cd8-(concentrate)-76631<br>https://www.agilent.com/en/product/immunohistochemistry/visualization-systems/envision-flex-systems/envision-flex-mouse-high-ph-%28link%29-76775
